# Integrated compact regulators of protein activity enable control of signaling pathways and genome-editing in vivo

**DOI:** 10.1038/s41421-023-00632-1

**Published:** 2024-01-23

**Authors:** Nik Franko, António José da Silva Santinha, Shuai Xue, Haijie Zhao, Ghislaine Charpin-El Hamri, Randall Jeffrey Platt, Ana Palma Teixeira, Martin Fussenegger

**Affiliations:** 1 Department of Biosystems Science and Engineering, ETH Zurich, Basel, Switzerland; 2 Département Génie Biologique, Institut Universitaire de Technologie, Université Claude Bernard Lyon 1, Villeurbanne, Cedex France; 3 Faculty of Science, University of Basel, Basel, Switzerland

**Keywords:** Biological techniques, Molecular biology

## Abstract

Viral proteases and clinically safe inhibitors were employed to build integrated compact regulators of protein activity (iCROP) for post-translational regulation of functional proteins by tunable proteolytic activity. In the absence of inhibitor, the co-localized/fused protease cleaves a target peptide sequence introduced in an exposed loop of the protein of interest, irreversibly fragmenting the protein structure and destroying its functionality. We selected three proteases and demonstrated the versatility of the iCROP framework by validating it to regulate the functional activity of ten different proteins. iCROP switches can be delivered either as mRNA or DNA, and provide rapid actuation kinetics with large induction ratios, while remaining strongly suppressed in the off state without inhibitor. iCROPs for effectors of the NF-κB and NFAT signaling pathways were assembled and confirmed to enable precise activation/inhibition of downstream events in response to protease inhibitors. In lipopolysaccharide-treated mice, iCROP-sr-IκBα suppressed cytokine release (“cytokine storm”) by rescuing the activity of IκBα, which suppresses NF-κB signaling. We also constructed compact inducible CRISPR-(d)Cas9 variants and showed that iCROP-Cas9-mediated knockout of the PCSK9 gene in the liver lowered blood LDL-cholesterol levels in mice. iCROP-based protein switches will facilitate protein-level regulation in basic research and translational applications.

## Introduction

Manipulation of macromolecules to achieve user-defined control over biological systems is a central goal of synthetic biology, and synthetic transcriptional circuits have been widely used for controlling protein levels to achieve desired cellular behavior in response to external cues^1^. Indeed, fine-tuning of synthetic transcription factors^2^ has enabled cells to exhibit complex computer-like behavior^3–6^. In addition, transcriptional regulation has been considered for an increasing number of therapeutic applications, such as inducible expression of chimeric antigen receptors in CAR-T cells for cancer treatment^7^, or expression of other therapeutic proteins in response to exogenous signals^8,9^ or endogenous disease biomarkers^10–13^. However, there are two major limitations to this approach: (i) the need to co-express regulatory components increases the genetic footprint and thus the complexity of the whole system, and (ii) the slow activation kinetics, together with long reversibility cycles, contributes to poor temporal resolution.

These issues can be overcome by directly controlling the protein of interest (POI) at a post-translational level, but this requires modifications of the target protein itself. So far, the most general strategy for direct regulation of protein function is based on dimerization systems that regulate the reconstitution of the target protein through light^14,15^, or small-molecule-induced protein proximity^16^. Chemical stimuli have advantages over physical stimuli for in vivo applications, including precise dosing, simple administration, systemic delivery (availability) and deep tissue penetration. Classical chemical-induced dimerization (CID) systems responsive to rapamycin^17^, abscisic acid^18^ and gibberellic acid^19^ have found many synthetic biology applications, including regulation of transcription^20^, genome modification^21^, proteolysis^22^, protein secretion^23,24^, chimeric antigen receptor formation^25^, post-translation-based logic operations^26,27^ and induction of apoptosis^28^. More recently introduced CID systems regulate dimerization via hormones such as T3 and cortisol^29^ or clinically approved drugs such as dasatinib^29^ or grazoprevir^30^. However, CID systems require co-expression of two polypeptide chains to control one output, which increases the genetic footprint, and also require fine-tuning for optimal activity. Consequently, simultaneous regulation of multiple functions with a single input is difficult. Moreover, since many CID systems rely on mammalian-derived components, small molecules controlling proximity can interact with native cellular machinery or cause unwanted side effects^31^, imposing limitations on their wider use.

To overcome these difficulties, we set out to develop protein switches consisting of single-chain proteins with built-in regulatory components, based on viral proteases for which potent inhibitors with a demonstrated safety profile in human clinical trials are available. Although this type of regulation appears to have great potential, only a limited number of hepatitis C virus protease (HCVp)-engineered proteins have so far been reported^32–34^, and robust design principles are lacking. Here, we propose a versatile design framework called integrated compact regulators of protein activity (iCROP), which can be used to control single- or multi-domain proteins. Briefly, target proteins harboring a protease cleavage site in a permissible region (usually an exposed loop) are cleaved into two inactive parts by default by the attached protease, but remain intact and active in the presence of protease inhibitors. As viral proteases, we focused on HCVp, human immunodeficiency virus protease (HIVp) and human rhinovirus protease (HRVp). Among the three proteases, HRVp with its inhibitor rupintrivir afforded the best-performing protein switches, exhibiting lower basal activity and higher activation rate than the widely employed HCVp.

In order to validate our design strategy, we first showed that the iCROP framework could deliver robust and rapid activation of ten functional proteins with large induction ratios, while remaining strongly suppressed in the off state (in the absence of inhibitor). We further established that iCROPs for effectors of NF-κB and NFAT signaling pathways enabled precise activation/inhibition of downstream events in response to the protease inhibitors. Notably, iCROP-sr-IκBα repressed cytokine release in lipopolysaccharide (LPS)-treated mice by rescuing the activity of IκBα, which suppresses NF-κB signaling. We also constructed extremely compact inducible CRISPR-(d)Cas9 variants, and showed that iCROP-Cas9-mediated knockout of the *PCSK9* gene in the liver lowered the blood LDL-cholesterol levels in mice. These results suggest that the iCROP system should be applicable for protein-level regulation in translational applications.

## Results

### Design of small-molecule-responsive regulators of protein activity

**Fig. 1:**
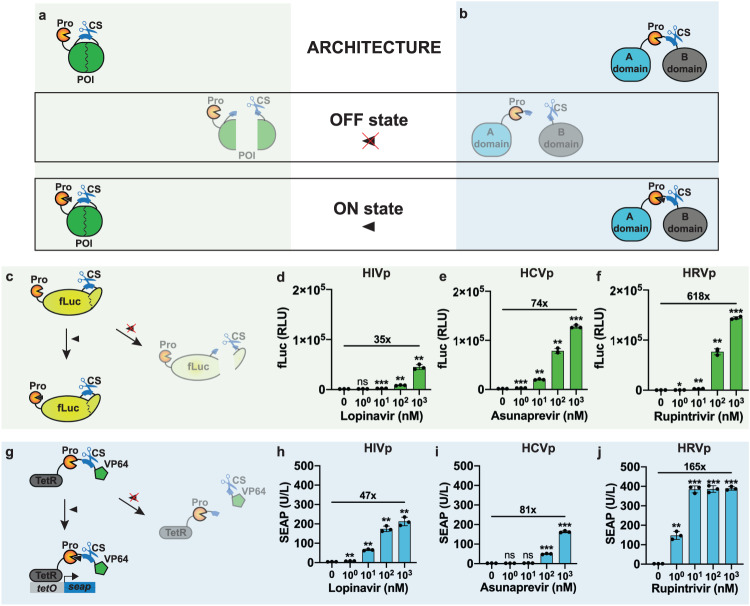
Compact small-molecule-responsive protein switches. **a**, **b** Schematic representation of the protein switches. The POI is modified to contain a protease cleavage site (CS) in a permissible location, and the corresponding protease is either **a** fused to the N-terminus or **b** placed next to the CS, between two domains (A and B) of the POI. In both configurations, the protease inactivates the functionality of the effector protein (OFF state). The addition of a small molecule inhibitor blocks the protease activity, thereby preserving the activity of the effector protein (ON state). **c** Scheme of synthetic post-translational regulation of fLuc. The protease CS was placed downstream of fLuc residue K491 and the protease from **d** HIV, **e** HCV, or **f** HRV was fused at the N-terminus. **d**–**f** Bioluminescence intensity of HEK293T cells transfected with each fLuc protease system and treated for 24 h with different concentrations of the corresponding inhibitors. **g** Scheme of synthetic post-translational regulation of the transcription factor TetR-VP64. The protease from **h** HIV, **i** HCV, or **j** HRV and cognate CSs were placed between TetR and VP64. **h**–**j** SEAP secretion from HEK293T cells co-transfected with each TetR-VP64 protease system and a tetO-driven SEAP reporter, and treated for 24 h with different concentrations of the corresponding inhibitors. In **d**, **e**, **f**, **h**, **i**, **j** data are shown as mean ± SD, with individual data points (*n* = 3 biological replicates). Numbers above bars indicate fold difference between values at the highest concentration and un-induced cells. Statistical significance was calculated by means of Welch’s two-tailed *t*-test, *P < 0.05, **P < 0.01, ***P < 0.001, ****P < 0.0001, ns, not significant.

To develop compact post-translational regulatory systems responsive to small molecules, we harnessed viral proteases whose activity can be inhibited by clinically safe compounds. We explored two routes to achieve regulation of any POI. A POI is modified to contain a protease cleavage site (CS) within its structure and the corresponding protease is placed either at its N- or C-terminus (Fig. 1a), or adjacent to the CS, separating two functional domains of the POI (Fig. 1b). In both configurations, the activity of the target protein is hijacked by the protease, which splits the POI into two fragments, destroying its functionality (OFF state). In the presence of the appropriate protease inhibitor, the protease cannot cleave the POI, which remains active (ON state). Several criteria must be met when engineering the POI. The CS should be placed into a permissible site, preferably an exposed loop, to facilitate the access to the protease without severely impacting the POI functionality. Likewise, the protease insertion into the POI structure or at one of its ends should not have a negative effect on the POI activity. Furthermore, to achieve low basal activity in the OFF state, the POI should be inactive upon cleavage, i.e., neither of the two split parts/domains can retain activity on its own or interact after the cleavage to reconstitute POI functionality. In order to build protein switches with translational potential, we tested the proposed framework with viral proteases from HIV, HCV and HRV (Supplementary Table S1), in combination with potent inhibitors that are either clinically approved or have shown favorable safety profiles in phase I clinical trials. We focused on these proteases because of their relatively small size, which helps to keep the genetic footprint of the full protein switch to a minimum, as well as their high catalytic activity, which is crucial to achieve low basal POI activity in the OFF state and high-fold activation in the ON state.

Initially, we tested post-translational regulatory switches based on each protease with two POIs whose activity is easily measured, namely the firefly luciferase (fLuc) (Fig. 1c–f) and the synthetic transcription factor TetR-VP64 (Fig. 1g–j), which activates the expression of the reporter protein secreted alkaline phosphatase (SEAP) downstream of a TetR-responsive promoter. FLuc was modified by fusing each protease to its N-terminus and placing the corresponding CS (Supplementary Table S2) downstream of the fLuc residue K491, which had been previously used as a split site for fLuc^26^ (Fig. 1c). HEK293T cells expressing each protein switch and treated with varying concentrations of the corresponding inhibitors showed a dose-dependent increase in the fLuc signal for all protease systems (Fig. 1d–f), with each system remaining unresponsive to the other two protease inhibitors (Supplementary Fig. S1). However, the degree of fLuc activation was protease-dependent, and the highest fold-increase was achieved with HRVp (Fig. 1f), followed by HCVp (Fig. 1e). Combining all three protease-regulated fLuc systems enabled us to engineer 2- and 3-input OR logic gates (Supplementary Fig. S2). For TetR-VP64, we placed the CSs in the linker between TetR and VP64, and the proteases either at the N-terminus of TetR (Supplementary Fig. S3a–d) or immediately upstream of the CS (Fig. 1g–j). While HIVp or HCVp placed between TetR and VP64 rather than at its N-terminus provided considerably higher activation efficiencies in the presence of the corresponding inhibitors (Fig. 1h, i; Supplementary Fig. S3b, c), the switches based on HRVp showed robust performance in both configurations (Fig. 1j; Supplementary Fig. S3d), and was significantly greater than those of the switches based on the other two proteases. Furthermore, stable integration of the switch based on HRVp (configuration in Supplementary Fig. S3a) in the genome of HEK293T cells resulted in the retention of robust and consistent performance throughout multiple passages (Supplementary Fig. S3e, f). The C-terminus of HRVp acts as a CS and deletion of the last amino acid (Q182) allows the chimeric transcription factor TetR-HRVp_ΔQ182_-VP64 to remain active even in the absence of the HRVp inhibitor (Supplementary Fig. S3g). In contrast, the introduction of a CS (Supplementary Table S2) enables cleavage and inactivation of the transcription factor (Supplementary Fig. S3g). Compared to the digital on/off-like behavior of the wild-type HRVp (Supplementary Fig. S3h), this version of the TetR-VP64 switch provides a more graded response to rupintrivir (Supplementary Fig. S3i). While introducing either the protease or the CS alone had marginal effect on the TetR-VP64 activity (Supplementary Fig. S4a), fLuc showed higher susceptibility to these modifications (Supplementary Fig. S4b).

Since HRVp was the best performing among the proteases for both POI configurations, it was selected for incorporation into all further protein switches developed in this work. To showcase that single-domain POIs are less likely to be suitable for protease insertion within the structure (Supplementary Fig. S5a), we placed HRVp upstream of its CS in fLuc and tested the construct in HEK293T cells treated with different doses of rupintrivir (Supplementary Fig. S5b). Although a 4.6-fold activation was obtained at the maximum non-toxic concentration tested (10 μM) relative to non-treated cultures (Supplementary Fig. S5b), even providing the HRVp in trans (expressed from a different plasmid, not fused to fLuc) resulted in a 22-fold increase in fLuc signal (Supplementary Fig. S5c), which is still greatly inferior to the over 500-fold luminescence increase obtained for HRVp fused to the fLuc N-terminus (Fig. 1f; Supplementary Fig. S5d). Therefore, as a rule of thumb for incorporating small-molecule-responsive post-translational regulation into POIs, the protease should preferentially be placed at one POI terminus for single-domain proteins, or between two domains for multi-domain proteins. We designated the developed protein switches, relying on inducible inactivation of POI-attached viral proteases, as integrated compact regulators of protein activity (iCROP).

### Characterization of iCROP kinetics and reversibility

**Fig. 2:**
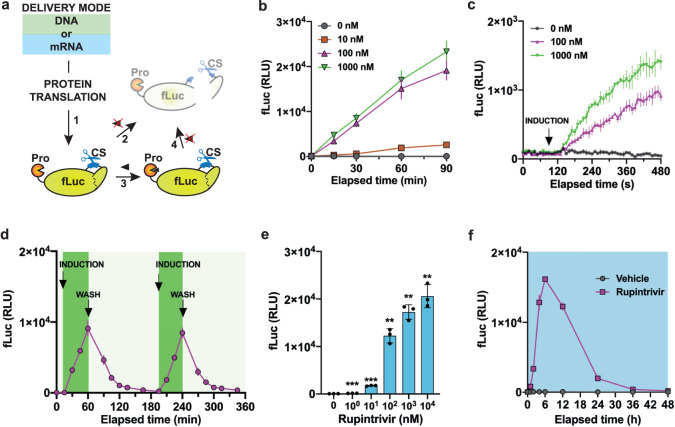
Kinetics and reversibility of iCROP-modified fLuc delivered as DNA or mRNA. **a** Schematic representation of the post-translational steps that iCROP-fLuc undergoes in the presence or absence of the protease inhibitor. **b** FLuc luminescence every 15 to 30 min during 90 min of incubation with rupintrivir at the indicated concentrations. HEK293T cells were transfected with iCROP-fLuc (P_mPGK_-HRVp_ΔQ182_-fLuc_CS-K491_-pA) and 36 h later the medium was exchanged for fresh medium either without or with rupintrivir. **c** Real-time fLuc analysis from HEK293T cells expressing iCROP-fLuc. The recording of the signal from live cells was started 90 s before changing the medium to fresh medium either without or with rupintrivir (100 nM or 1000 nM). **d** iCROP-fLuc reversibility. HEK293T cells expressing iCROP-fLuc were alternated between rupintrivir-containing (100 nM) and rupintrivir-free media. Each rupintrivir induction lasted 45 min, followed by 135 min in rupintrivir-free medium. fLuc intensity was measured at the indicated time points. **e** Inducibility of iCROP-fLuc delivered as mRNA. Luminescence from HEK293T cells transfected with in vitro-transcribed mRNA coding for iCROP-fLuc for 6 h, followed by incubation for additional 6 h in the presence of the indicated rupintrivir concentrations. **f** Activation kinetics of mRNA-delivered iCROP-fLuc. HEK293T cells simultaneously transfected with iCROP-fLuc mRNA and treated with rupintrivir (1 μM) were analyzed for fLuc intensity at the indicated time points. In **b**–**f**, data are shown as mean ± SD (*n* = 3 biological replicates). Statistical significance was calculated by means of Welch’s two-tailed *t*-test, *P < 0.05, **P < 0.01, ***P < 0.001, ****P < 0.0001, ns, not significant.

Different from most common protein switches, which rely on an inducer to bring two (or more) inactive protein split domains together for activation upon translation^16,35^, our system relies on the catalytic activity of a protease which is active immediately after translation (Fig. 2a, step 1). For a robust protein switch performance, the self-inactivation process (step 2) needs to be rapid and efficient to ensure minimal basal POI activity, but with just enough lag time between step 1 and 2 to prevent the proteolysis of the POI (step 3) when the input molecule is present. Ideally, a rapid decrease in POI activity should occur after removing the input (step 4) to enable system reversibility and repeated activation.

To characterize the activation kinetics, we measured fLuc every 15 to 30 min during 90 min after addition of the protease inhibitor and observed a dose-dependent increase in fLuc signal within minutes (Fig. 2b). After 15 min incubation the fold-changes at 100 nM or 1 μM inhibitor relative to non-treated cultures were already 41× and 57×, respectively. Extending the monitoring period to 48 h after treatment increased the fold-difference to 1401× at 1 μM (Supplementary Fig. S5e). Furthermore, to achieve a more detailed characterization of the activation kinetics, we followed the luminescence signal in real-time in cellulo immediately after adding rupintrivir (Fig. 2c). The fLuc signal increased within seconds of treatment with rupintrivir (1 μM), indicating that this type of protein regulation retains rapid activation kinetics. For the reversibility experiment, we pulsed the rupintrivir input and were able to repeatedly regulate fLuc activity and perform multiple OFF-ON-OFF cycles within 6 h (Fig. 2d) or 24 h (Supplementary Fig. S5f), depending on the pulse duration, with low leakiness and high activation levels.

Next, we characterized the response of the fLuc proteolysis device delivered as mRNA rather than DNA. Cells transfected with mRNA and treated for 6 h with different input concentrations showed a dose-dependent increase in fLuc luminescence (Fig. 2e), reaching high fold-changes relative to non-treated cultures, with sensitivity similar to that observed after DNA-based delivery. The time course of the fLuc signal showed a fast increase in luminescence, starting immediately after mRNA delivery, for rupintrivir-treated cultures, peaking at ~6 h post transfection, even though the inducer was not washed out (Fig. 2f). This presumably reflects the shorter half-life of mRNA-delivered protein switches, which would be useful for some applications. At the same time, mRNA delivery avoids the risks associated with random DNA integration into the host genome. Despite the inherent instability of the iCROP-fLuc transcripts, we were still able to observe an ON-OFF-ON-OFF cycle in response to shorter induction periods (90 min) with rupintrivir (Supplementary Fig. S6).

### Regulation of endogenous signaling pathways

We next sought to apply the iCROP system to establish control over proteins involved in endogenous signaling pathways in order to enable orthogonal small molecule-responsive activation or repression of target pathways. We first focused on engineering inducible proteins that serve as signaling mediators in the NF-κB pathway, namely MyD88, RelA and IκBα.

**Fig. 3:**
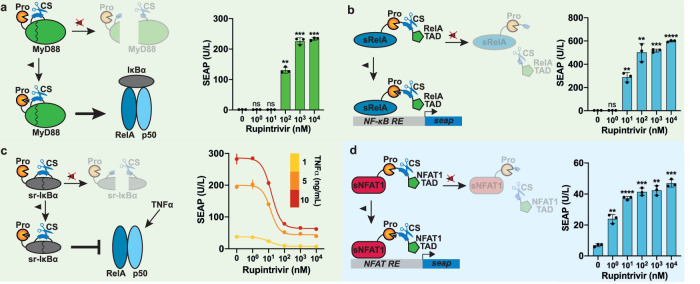
Regulation of endogenous signaling pathways by iCROP-engineered signaling mediators. **a** MyD88-mediated NF-κB activation in response to rupintrivir, which preserves the function of iCROP-engineered MyD88. MyD88 bearing the HRV protease at its N-terminus and the cognate CS at position G83 is fragmented into two inactive parts unless the protease inhibitor is present. When rupintrivir is present, intact MyD88 activates downstream effectors, culminating with the assembly of the complex in the promoter region of NF-κB target genes (left). HEK293T cells co-transfected with constitutive expression of iCROP-MyD88 (P_mPGK_-HRVp_ΔQ182_-Myd88_CS-G83_-pA) and SEAP expression from an NF-κB-responsive promoter (P_NF-κB_-SEAP-pA) were treated with rupintrivir at the indicated concentrations for 24 h before analyzing SEAP expression levels (right). **b** RelA-mediated NF-κB activation in response to rupintrivir, which preserves the function of iCROP-engineered RelA (left). RelA bearing both the HRVp and CS at position S319 is fragmented into two non-functional parts, unless the protease inhibitor is provided, when transcription of the reporter SEAP can occur. HEK293T cells were co-transfected with constitutively expressed iCROP-RelA (P_mPGK_-N-RelA-HRVp-RelA-C-pA) and P_NF-κB_-SEAP-pA and treated with rupintrivir at various concentrations for 24 h before analyzing SEAP expression levels (right). **c** Super-repressor sr-IκBα-mediated NF-κB repression in response to rupintrivir. sr-IκBα bearing the HRV protease at its N-terminus and the cognate CS at position P170 is fragmented into two inactive parts, unless the protease inhibitor is provided, thereby suppressing NF-κB signaling (left). HEK293T cells co-transfected with iCROP-sr-IκBα and P_NFκB_-SEAP-pA were challenged with the indicated TNFα concentrations to induce NF-κB signaling and treated with different doses of rupintrivir for 24 h before analyzing SEAP expression levels (right). **d** NFAT1-mediated activation of NFAT signaling in response to rupintrivir. The HRVp and CS were placed between the truncated DNA-binding domain (sNFAT1_S172-G701_-DBD) and the transactivation domain (NFAT1_L702-T925_-TAD) (left). HEK293T cells co-transfected with iCROP-NFAT1 and SEAP expression from an NFAT-responsive promoter (pMX57, P_3xNFAT_-SEAP-pA) were treated with the indicated rupintrivir concentrations for 24 h before analyzing SEAP expression levels (right). Data are shown as mean ± SD (*n* = 3 biological replicates) in all panels. Statistical significance was calculated by means of Welch’s two-tailed *t*-test, *P < 0.05, **P < 0.01, ***P < 0.001, ****P < 0.0001, ns, not significant.

MyD88 mediates the signal transduction between toll-like receptors (TLRs) and downstream signaling effectors that result in NF-κB activation^36^. To engineer an inducible MyD88, we examined its 3D structure (Supplementary Fig. S7a) and selected three possible locations to place the CS, namely after G83, R188 and G201. We then tested the effect of introducing the CS at each location on the protein activity by co-transfecting each MyD88 variant together with an NF-κB-responsive promoter driving SEAP expression. We detected increased SEAP expression in cells engineered with each MyD88 variant, albeit to slightly lesser extent than in the case of the wild-type protein (Supplementary Fig. S7b). Next, we fused the HRVp to the N-terminus of each MyD88 variant and tested how efficiently the fusions could be proteolytically inactivated and their activity rescued by rupintrivir. The MyD88_CS-G83_ variant was the most efficiently fragmented by the attached HRVp, with a large decrease in SEAP expression (very low leakiness) relative to the same variant in the absence of the protease (Supplementary Fig. S7c). The other two CS locations might be less accessible to the fused protease or the resulting fragments still might have some activity, since only around a 2-fold increase in SEAP expression was observed between rupintrivir-treated and non-treated cultures (Supplementary Fig. S7c). MyD88_CS-G83_ showed rupintrivir responsiveness, with a more than 150-fold increase in SEAP secretion, thus confirming the feasibility of controlling the NF-κB pathway with our framework (Fig. 3a). Alternatively, we took RelA (also known as p65), which acts downstream of MyD88, as another possible effector protein to establish inducible activation of the NF-κB signaling pathway. HRVp with its CS was placed between the DNA-binding domain of RelA (RelA-DBD) and its transactivation domain (RelA-TAD) (Supplementary Fig. S7d). The generated protein self-inactivates very efficiently upon translation, as suggested by the very low SEAP expression from the NF-κB-responsive promoter. Rupintrivir rescued RelA activity, increasing SEAP expression in a dose-dependent manner (Fig. 3b). In addition, the sensitivity to the protease inhibitor could be adjusted by using the HRVp_ΔQ182_ variant together with the CS (Supplementary Fig. S7e).

Finally, to establish inducible repression of NF-κB signaling we engineered the stable super-repressor form of IκBα (sr-IκBα), which is a double-mutant IκBα that has been shown to block NF-κB activation when over-expressed in cells^37^. Three locations in the sr-IκBα structure (Supplementary Fig. S7f) appeared suitable for the CS, while the HRVp was placed at the N-terminus. We first confirmed that the addition of the ligand tumor necrosis factor (TNFα) leads to activation of NF-κB signaling in a dose-response manner, in cells transfected with the NF-κB reporter construct (Supplementary Fig. S7g). The three CS-modified sr-IκBα variants were compared to non-modified sr-IκBα in terms of their ability to suppress NF-κB signaling in TNFα-treated cells. The sr-IκBα_CS-P170_ variant suppressed NF-κB with the highest efficiency, being only slightly less effective than the non-modified variant (Supplementary Fig. S7h). The fusion of HRVp to sr-IκBα_CS-170_ allowed dose-dependent control of its activity by rupintrivir, as monitored by the decrease in SEAP expression from the NF-κB-responsive promoter, demonstrating titratable NF-κB signaling repression across different levels of TNFα-activated NF-κB signaling (Fig. 3c).

Next, we focused on engineering members of the nuclear factor of activated T cells (NFAT) transduction pathway, namely NFAT1 and NFAT2 (Supplementary Fig. S8a). First, we truncated the N-terminal part of NFAT1 to create a more compact version and embedded the wild-type HRVp between the shortened DNA-binding module (sNFAT1_S172-G701_-DBD) and the transactivation module (NFAT1_L702-T925_-TAD). The resulting protein exhibited much lower activity than the wild-type NFAT1 as measured in terms of SEAP expression from the NFAT-responsive promoter in co-transfection experiments with each NFAT1 variant (Supplementary Fig. S8b). Part of the activity of the modified NFAT1 could be rescued by adding the protease inhibitor, demonstrating a titratable NFAT activation in response to rupintrivir (Fig. 3d). To test whether the performance could be improved, we replaced the native transactivation module by VP64 (Supplementary Fig. S8c) and used the HRVp_ΔQ182_ variant together with the CS; these changes decreased the basal activity and increased the fold-changes in response to rupintrivir (Supplementary Fig. S8d). The same configuration was applied to engineer NFAT2, which also afforded robust rupintrivir-inducible activation of NFAT signaling (Supplementary Fig. S8e). The functionality of both NF-κB (Supplementary Fig. S9a) and NFAT (Supplementary Fig. S9b) effectors was additionally confirmed in Jurkat T cells. Finally, we demonstrated that by co-expressing protease-engineered effectors of both pathways, we can simultaneously control NF-κB and NFAT signaling, either activating both (Supplementary Fig. S10a) or activating NFAT and repressing NF-κB (Supplementary Fig. S10b) with a single input. Orthogonal regulation was further showcased by independently controlling three different proteins without any crosstalk (Supplementary Fig. S11).

### Regulation of endogenous gene expression and genome editing with inducible (d)Cas9

**Fig. 4:**
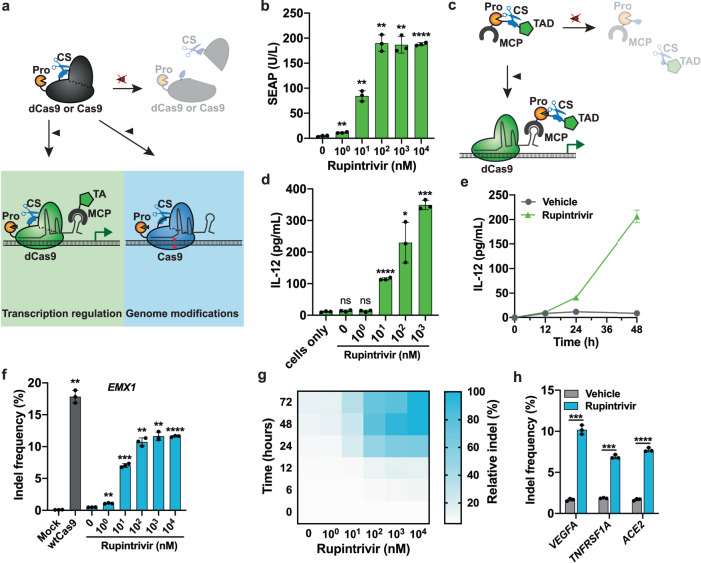
Transcriptional regulation and genome editing using iCROP-engineered (d)Cas9 protein or MCP_VP64_. **a** Schematic representation of the iCROP-modified (d)Cas9 protein. dCas9 or Cas9 protein bearing the HRV protease at the N-terminus and the cognate CS at position R535 is fragmented into two inactive parts, unless the protease inhibitor is present. In the presence of inhibitor, (i) active dCas9 associates with a target sgRNA fused to an MS2 loop, which co-localizes the MCP protein fused to a transcriptional activation domain in the promoter region of the target gene, thereby activating transcription; and (ii) active Cas9 associates with a target sgRNA introducing mutations in a target genomic locus. **b** SEAP produced by cells constitutively expressing iCROP-dCas9 (P_mPGK_-HRVp_ΔQ182_-dCas9_CS-R535_-pA), MS2 loop fused to an sgRNA targeting the insulin promoter (P_U6_-sgRNA(P_INS_)-MS2), MCP fused to the transactivation domain (pKK44, P_hCMV_-MCP-p65_TA_-HSF1_TA_-pA) and SEAP expression under an insulin-responsive promoter (P_INS_-SEAP-pA), and treated for 24 h with the indicated concentrations of rupintrivir. **c** Schematic illustration of the iCROP-MCP_VP64_ system for dCas9-guided transcription regulation in response to rupintrivir. **d** Activation of gene expression from the endogenous genomic target with the iCROP-MCP_VP64_ system. Cells were co-transfected with dCas9, iCROP-MCP_VP64_ and sgRNA targeting the IL-12B promoter (sgRNA(P_IL-12B_)-MS2) and treated with the specified rupintrivir concentrations for 48 h before analyzing IL-12 levels in the supernatant by ELISA. **e** Kinetics of iCROP-MCP_VP64_-based activated IL-12 expression. Time-course analysis of IL-12 levels in culture supernatants from cells treated either with 1 µM rupintrivir or vehicle. **f** Mutation rate in the *EMX1* gene in cells expressing iCROP-Cas9 and sgRNA(EMX1), and treated with indicated concentrations of rupintrivir for 48 h before NGS analysis. Cells co-transfected with non-modified Cas9 and sgRNA(EMX1) were included as a positive control. **g** Heat-map representing time- and dose-dependent *EMX1* relative indel frequency for iCROP-Cas9. Relative indel frequency was calculated as the ratio between the mutation rate at each specified dose-time point and the maximal mutation rate (*n* = 3 biological replicates). **h** Frequency of indel mutations in the genomic loci of *VEGFA*, *TNFRSF1A* and *ACE2* genes in cells co-transfected with iCROP-Cas9 and each sgRNA targeting gene, and kept for 48 h in the absence or presence of rupintrivir (1 μM) (sgRNA variants with the highest fold induction of mutation rates are represented (see Supplementary Fig. S12g for efficiency of other sgRNA). In panels **b**, **d**, **e**, **f**, **h**, data are shown as mean ± SD (*n* = 3 biological replicates). Statistical significance was calculated by means of Welch’s two-tailed *t*-test, *P < 0.05, **P < 0.01, ***P < 0.001, ****P < 0.0001, ns, not significant.

CRISPR-Cas9 based technology has revolutionized the field of genome engineering in the last decade. Several inducible (d)Cas9-based systems have been reported, but they suffer from a large genetic footprint^38^, high basal activity when expressed transiently^21^ or use unfavorable compounds for activation^39^. Therefore, we decided to engineer compact inducible (d)Cas9 systems for transcription regulation and genome modifications by applying the iCROP framework (Fig. 4a). We first identified three potential permissible sites within the dCas9 structure to place the protease CS (Supplementary Fig. S12a). The activity of each CS-modified dCas9 was tested by co-transfecting HEK293T cells with a validated sgRNA targeting the human insulin promoter^40^ attached to an MS2 loop (sgRNA(P_INS_)-MS2), the MS2-coat protein (MCP) fused to a transcription activation domain, and the human insulin promoter driving SEAP expression (P_INS_-SEAP-pA). SEAP expression could be activated with all dCas9 variants, but the introduction of the CS seemed to have affected their activity to different extents, with reductions of 20%, 7% and 42% for CS_E311_, CS_R535_ and CS_V713_, respectively, relatively to that achieved with the non-modified dCas9 (Supplementary Fig. S12b). Moreover, the fusion of HRVp to CS-modified dCas9 proteins drastically reduced SEAP expression, suggesting inactivation of dCas9 by cleavage. SEAP expression could be dose-dependently activated by rupintrivir for all iCROP-modified dCas9 variants (Fig. 4b; Supplementary Fig. S12c, d), confirming the feasibility of tuning dCas9 activity by varying the input concentration. dCas9_CS-R535_ was the best performing variant (Fig. 4b), with lower basal activity and higher SEAP transcription activation than the other two variants (Supplementary Fig. S12c, d). Alternatively, we were also able to engineer the MCP-VP64 component of the dCas9-based transcription regulation system, by placing the HRV protease and cognate CS between the two domains (Fig. 4c), achieving rupintrivir-inducibility with different sensitivities, depending on the applied protease and CS module (Supplementary Fig. S12e, f). To showcase transcriptional regulation of endogenous genes, we targeted the iCROP-engineered dCas9 system to the genomic promoter region of the *IL12B* gene. We were able to achieve high activation of IL-12 expression in cells treated with different rupintrivir concentrations, obtaining a linear increase of protein production over the concentration range of 10 nM to 1 μM of the input (Fig. 4d) with negligible leakiness. IL-12 accumulation in the culture supernatant increased throughout the analyzed 48 h period (Fig. 4e).

To establish rupintrivir-inducible genome editing, we engineered Cas9 with the iCROP system that performed best for dCas9. Co-expression of the iCROP-modified Cas9 and an sgRNA targeting the *EMX1* gene in HEK293T cells treated with rupintrivir resulted in an increasing number of indel formation in a rupintrivir-dependent manner, as confirmed by next-generation sequencing (NGS) analysis of isolated genomic DNA (Fig. 4f). Indeed, self-cleaved iCROP-Cas9 exhibited low basal activity in the absence of rupintrivir and could be activated at 1 nM rupintrivir, with a maximal indel frequency at 1 μM, which is only 1.53-fold lower than what was obtained with the wild-type Cas9. We then analyzed the indel frequency for different incubation periods and different doses of rupintrivir. Cells treated with 1 or 10 μM rupintrivir showed iCROP-mediated indels as early as 6 h post-induction (Fig. 4g). The greatest increase in indel formation was observed between 12 h and 24 h of rupintrivir treatment, for all tested concentrations.

Lastly, we validated rupintrivir-inducible genome editing for three additional human genes, namely *VEGFA*, *TNFRSF1A*, and *ACE2* (Fig. 4h). By designing and testing three different sgRNAs for each gene, we were able to install indels in all three genomic loci in rupintrivir-treated cells, with different indel mutation rates that were dependent on the sgRNA sequence (Supplementary Fig. S12g).

### Inducible suppression of cytokine storm and activation of genome editing in mice

**Fig. 5:**
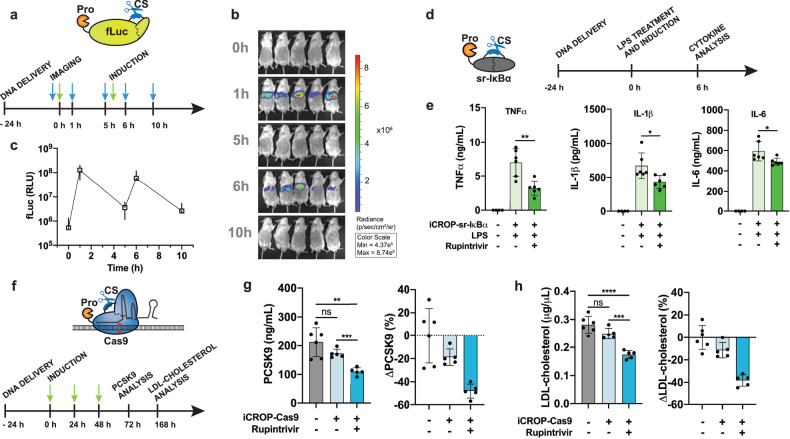
Activity and functional performance of iCROP systems in vivo. **a** Timeline for iCROP-fLuc study in vivo. Mice were hydrodynamically injected with the plasmid encoding iCROP-fLuc 24 h before rupintrivir administration. **b** Luminescence images were recorded at the indicated time points by the IVIS imaging system (*n* = 5 mice). **c** Quantification of the luminescence intensity of images in **b**. **d** Timeline for the in vivo study using iCROP-sr-IκBα to repress NF-κB signaling. The mice received the iCROP-sr-IκBα-encoding plasmid by hydrodynamic injection 24 h before the LPS treatment and rupintrivir administration. Blood samples were collected for cytokine analysis 6 h later. **e** Blood levels of TNFα, IL-1β and IL-6 6 h after the LPS treatment, analyzed by ELISA. Data are shown as mean ± SD, with individual data points (*n* = 6 mice per experimental group, *n* = 4 mice for wild-type group). **f** Timeline for the in vivo study using inducible iCROP-Cas9 for *PCSK9* knockout. The DNA vectors expressing iCROP-Cas9 and sgRNA(mPCSK9) were delivered to mice 1 day before the first rupintrivir treatment, which was repeated for 3 consecutive days, before analysis. **g** Serum PCSK9 levels measured by ELISA (left) in wild-type mice, mice expressing only the iCROP-Cas9 system and mice expressing the iCROP-Cas9 system in combination with rupintrivir treatment. Results are shown as absolute PCSK9 values (left) and relative PCSK9 reduction compared to wild-type mice (right). Relative decrease was calculated by dividing the average PCSK9 levels of experimental groups by the average PCSK9 level of the wild-type group. **h** LDL-cholesterol levels in mouse serum, measured with an LDL-cholesterol assay kit 168 h after starting the rupintrivir dosing (left) and relative LDL-cholesterol reduction compared to wild-type mice (right). Relative decrease was calculated by dividing the average LDL-cholesterol levels of experimental groups by the average LDL-cholesterol level of the wild-type group. In panels **g,** **h**, data are shown as mean ± SD, with individual data points (*n* = 5–6 mice per group). Statistical significance was calculated by means of Welch’s two-tailed *t*-test, *P < 0.05, **P < 0.01, ***P < 0.001, ****P < 0.0001, ns, not significant.

Next, we sought to apply the protein switches in vivo. We selected the iCROP-fLuc system to assess the feasibility of regulating protein activity in engineered mice by rupintrivir administration. Before targeting liver tissue in vivo, we first confirmed that this system is functional in the liver cell line HepG2 (Supplementary Fig. S13). The DNA vector for constitutive expression of the iCROP-modified fLuc was delivered to mice via hydrodynamic tail vein injection (Fig. 5a). Mice imaged 24 h post DNA delivery showed a low basal fLuc signal (Fig. 5b, c), which rapidly increased upon rupintrivir administration in the upper abdominal liver region, reaching 240-fold higher luminescence intensity 60 min after the treatment. The fLuc signal was decreased when we recorded new images 4 h after the treatment, likely due to rupintrivir clearance. When we examined repeated rupintrivir administration at the same time schedule used for luminescence imaging, we observed similar patterns of fLuc signal appearance and disappearance (Fig. 5b, c). These results confirmed that fLuc can be rapidly activated in vivo, enabling repeatable and precise temporal protein regulation that appears to depend on rupintrivir’s pharmacokinetic properties.

Next, we tested the ability of the iCROP-sr-IκBα switch to repress cytokine release caused by LPS treatment (Fig. 5d). Mice engineered with iCROP-sr-IκBα and injected with LPS were treated either with rupintrivir or with vehicle only. Analysis of blood samples collected 6 h after LPS challenge showed 54%, 35%, and 18% reductions of TNFα, IL-1β, and IL-6 for the mouse group that received rupintrivir, compared to the group that received the vehicle (Fig. 5e). Rupintrivir alone did not have any effect on cytokine levels in LPS-treated mice lacking iCROP-sr-IκBα (Supplementary Fig. S14a–c). These results suggest that rupintrivir successfully rescued the activity of the super-repressor IκBα, which suppressed NF-κB signaling, thereby attenuating the production of cytokines in response to LPS.

We also assessed the suitability of iCROP-Cas9 for genome editing in vivo (Fig. 5f). We targeted the disruption of the proprotein convertase subtilisin kexin 9 (*PCSK9*) gene, whose protein is responsible for enhanced degradation of the low-density lipoprotein (LDL) receptor and thus reduced LDL-cholesterol clearance^41^. This is a promising therapeutic target to lower cholesterol levels, and a clinical trial using constitutive expression of the CRISPR/Cas9 components is currently in progress, though this system is prone to higher rates of off-target effects^42–44^, which compromises its safety profile. Here, after receiving the iCROP-Cas9 protein and the sgRNA targeting the murine *PCSK9* via hydrodynamic injection, mice were treated for three consecutive days with either rupintrivir or vehicle only. Serum PCSK9 levels (Fig. 5g) and LDL-cholesterol levels (Fig. 5h) were significantly decreased in engineered mice treated with rupintrivir when compared to non-treated mice or mice that did not receive the iCROP-Cas9 components, showing reduction of 48% and 38% for PCSK9 and LDL-cholesterol, respectively. We did not observe any changes in PCSK9 (Supplementary Fig. S15a) or LDL-cholesterol (Supplementary Fig. S15b) levels in mice that were treated with rupintrivir but lacked the iCROP-Cas9 components, compared to untreated mice. Additionally, mice that received iCROP-Cas9, with or without rupintrivir treatment, did not show elevated levels of inflammatory cytokines compared to wild-type mice (Supplementary Fig. S16a, b). These results demonstrate that iCROP-Cas9 is responsive to rupintrivir in vivo, and upon activation can target the loci of the host genome, highlighting the potential of our regulatable Cas9 technology to knock out genes in vivo in a defined temporal window.

## Discussion

We have developed a general design framework called iCROP for the construction of small-molecule-regulatable protein switches in mammalian cells. iCROP enables robust and rapid activation of a target protein with high sensitivity, a wide dynamic range and high temporal resolution. A key feature of iCROP is the compact design, which provides a small genetic footprint — it should be possible to pack most iCROP-controlled systems into viral vectors such as AAV. Furthermore, iCROP can be delivered as mRNA, which would avoid the risk of unwanted genomic integrations and be a useful feature when the POI has to be expressed only for a short duration. Importantly, we found that a wide range of proteins can accept a CS in their structure and a protease on the N-terminus with little impact on their functionality, suggesting broad applicability of the iCROP framework. In some cases, the protease could be inserted next to the CS between two protein domains as an alternative architecture for multi-domain proteins. Furthermore, in the case of proteins that have been previously engineered as split versions, the split location should also be permissible for the CS or both the CS and the protease. Recent developments in artificial intelligence (AI)-based tools and models for 3D protein structure prediction, such as AlphaFold^45^, should also be helpful to predict the most favorable CS location when de-novo engineering POIs for iCROP, analogously to computational tools for predicting the most favorable sites for protein splitting^46,47^.

We wish to emphasize that the iCROP system is different in principle from systems based on pre-split proteins, as it employs an intact molecule into which a CS has been introduced. Interestingly, although HCVp^33,34^ has been used in systems employing pre-split proteins, we found that HRVp afforded much lower leakiness and higher activation folds than HCVp in the iCROP system. In the future, it should be possible to incorporate other proteases, not limited to viral proteases, whose activity can be modulated with small molecule inhibitors in order to expand the iCROP toolbox and possibly achieve orthogonal control over multiple proteins simultaneously. Potential proteases and their inhibitors^48^ could be screened using technology recently developed by our group^49^. While the use of human-derived proteases would humanize the system, this would involve a tradeoff that inhibitors targeting the iCROP would likely have off-target effects and could interfere with homeostasis in vivo. Here, we used rupintrivir to control iCROP, but an improved version of the drug with good oral bioavailability^50^ that has been tested in humans at high doses without apparent side effects could be available for future in vivo applications.

Our results demonstrate the potential of the iCROP framework for a wide range of applications. Firstly, iCROPs for various effectors of NF-κB and NFAT signaling pathways enabled precise activation/inhibition of downstream events in response to protease inhibitors. Achieving control over cellular pathways by means of iCROP will be useful not only to study cell signaling but also to manipulate cells in vivo. In particular, to highlight the translational potential of iCROP, we showed that iCROP-sr-IκBα can suppress cytokine release in LPS-treated mice, serving as a “cytokine storm buster” by rescuing the activity of IκBα, which suppresses NF-κB signaling. This suggests that iCROP may be applicable to orthogonally up- or down-regulate specific pathways in therapeutic cells to improve the efficacy of cell therapies. In addition, we demonstrated the applicability of iCROP for gene editing — we constructed extremely compact inducible CRISPR-(d)Cas9 variants, and showed that iCROP-Cas9-mediated knockout of the *PCSK9* gene in mouse liver lowered the blood LDL-cholesterol levels. Thus, iCROP can be utilized to control the function of genome modifiers such as Cas9, providing a robust new tool for regulating or editing endogenous genes. We believe that the iCROP system will open up many new possibilities for protein-level regulation in basic research and translational applications.

## Materials and methods

### Plasmid construction

The design and cloning details for all genetic constructs used in the study are provided in Supplementary Tables S3–S5. Briefly, the constructs were generated by classic molecular biology approaches using restriction enzymes (New England BioLabs) followed by ligation using T4 DNA ligase (EL0011, Thermo Fisher Scientific). The PCR reactions were performed using Q5 High-Fidelity DNA polymerase (M0491L, New England BioLabs). All steps were performed according to the manufacturer’s instructions. The plasmids were amplified in *Escherichia coli* strain XL10-Gold® (XL10-Gold® ultracompetent cells; 200314, Agilent Technologies). Constructs were verified by Sanger sequencing, done by an external vendor (Microsynth AG). Synthetic gene fragments (Supplementary Table S6) used in the study were codon-optimized for expression in human cells and commercially synthesized (Twist Bioscience).

### Cell culture and transfection

Human embryonic kidney cells (HEK293T, ATCC: CRL-11268) and Hep G2 (HEPG2, ATCC: HB-8065) were cultured in Dulbecco’s modified Eagle’s medium (DMEM; 61965026, Thermo Fisher Scientific) supplemented with 10% (v/v) fetal bovine serum (FBS; F7524, Sigma-Aldrich) in a humidified atmosphere containing 7.5% CO_2_ at 37 °C. Passaging of pre-confluent HEK293T or HepG2 cells was done by detaching them through incubation in 2 mL of 0.05% trypsin-EDTA (25300054, Thermo Fisher Scientific) for 5 min at room temperature. Cells were transferred to 8 mL culture medium, centrifuged for 1 min at 200 × *g*, resuspended in fresh medium and re-seeded at the desired cell density. Quantification of cell number and viability was done by using a CellDrop automated cell counter (DeNovix).

### Transient transfection of mammalian cells

For transfection experiments, HEK293T cells were seeded into 96-well plates, either transparent (3599, Corning), black (655090, Greiner Bio-One) or white (655098, Greiner Bio-One) depending on the reporter used in the experiment at 2 × 10^4^ cells per well, 24 h before transfection. The transfection mixture for each well of 96-well plates consisted of 120 ng of plasmid DNA in 50 μL of FBS-free DMEM and 600 ng polyethyleneimine (PEI, MW 40,000; 24765, Polysciences). After 20 min incubation at room temperature, the transfection mixture was added dropwise to the cells, which were then incubated overnight. The next morning, the culture medium was replaced with fresh FBS containing DMEM with or without inducer, depending on the experiment, and the reporter expression was profiled 24 h later if not otherwise specified. For Cas9-related experiments, HEK293T cells were transfected with Lipofectamine 3000 Transfection Reagent (L3000001, Thermo Fisher Scientific) according to the protocol provided by the manufacturer. For transfection of HepG2 cells, they were seeded in black 96-well plates (655090, Greiner Bio-One) at 2 × 10^4^ cells per well, 24 h before transfection with Lipofectamine 3000 Transfection Reagent.

### Stable cell line generation

Polyclonal stable cell lines were generated by co-transfecting HEK293T cells with a Sleeping Beauty transposase (pTS395) expression vector in a 1:10 (w/w) ratio with pNF417, containing SB recognition sites and encoding a puromycin resistance marker and YPet fluorescent protein. The medium was exchanged 12 h after transfection and cells were incubated for 48 h before the addition of selection medium containing 2 µg/mL puromycin. Cells were maintained through passages in DMEM containing 10% FBS and 2 µg/mL puromycin.

### Jurkat T cell line electroporation and fluorescence intensity determination

Jurkat cells (Jurkat, Clone E60-1, ATCC: TIB152™) were cultivated in RPMI medium (cat. no. 72400-021; Thermo Fischer Scientific, Waltham, MA, USA) supplemented with 10% FBS, 100 U/mL penicillin and 100 µg/mL streptomycin. Electroporation was performed using a NEPA21 electroporator (Nepa Gene) with a total amount of 20 μg DNA. Cells were induced 24 h after electroporation with 1 μM rupintrivir and kept for a further 24 h before determining the mean fluorescence intensity (MFI) of citrine with a CytoFLEX S flow cytometer (Beckman Coulter).

### Inducer preparation

Rupintrivir (6414, Tocris or PZ0315, Sigma), lopinavir (7052, Tocris) and asunaprevir (HY-14434, MedChemExpress) were prepared as 20 mM solutions in DMSO. TNFα (300-01 A, PeproTech) was prepared as 1 μg/mL solution in FBS-free DMEM. Working solutions were prepared by serially diluting the stock solutions in FBS containing DMEM.

### Protein 3D structure visualization

Protein 3D structures were obtained from and visualized in Protein Data Bank (PDB).

### In vitro transcription and mRNA transfection

mRNA for transfection was produced by IVT reaction with a HiScribe T7 ARCA mRNA Kit (E2060S, New England BioLabs), according to the manufacturer’s instructions, using constructs under the T7 promoter as a template. A Monarch RNA Cleanup Kit (T2040S, New England BioLabs) was used to purify the mRNA product. mRNA transfection into mammalian cells was performed with Lipofectamine MessengerMAX Transfection reagent (LMRNA001, Thermo Fisher Scientific) according to the instructions provided by the manufacturer.

### FLuc quantification

FLuc bioluminescence intensity in cells was quantified with the ONE-Glo Luciferase Assay System (E6110, Promega). The supernatant was first aspirated from the cells, growing in either black (for experiments with plasmid DNA) or white (for experiments with IVT mRNA) 96-well plates, followed by addition of 100 μL ONE-Glo Reagent/PBS mixture (1:1). Plates with samples were incubated for 5 min at room temperature for cell lysis to occur and the luminescence intensity was measured with a Tecan Spark multiplate reader (Tecan AG).

### In cellulo real-time fLuc measurement

For the real-time in cellulo fLuc activation kinetics experiment, D-luciferin (LUCK-100, Gold Biotechnology) was added to the iCROP-fLuc-transfected cells to the final concentration of 1.5 mM. Plates were incubated for 30 min at 37 °C before addition of the inducer and then the luminescence intensity increase was immediately measured every 10 s for a total duration of 8 min with a Tecan Spark multiplate reader (Tecan AG).

### SEAP quantification

SEAP levels in cell culture supernatants were quantified by measuring the increase of absorbance due to hydrolysis of para-nitrophenyl phosphate (pNPP). A total of 40 μL of heat-inactivated (30 min, 65 °C) cell culture supernatant was transferred into a 96-well plate (260836, Thermo Fisher Scientific) and mixed with 60 μL water, 80 μL 2× SEAP buffer (20 mM homoarginine, 1 mM MgCl_2_, 21% (v/v) diethanolamine, pH 9.8) and 20 μL substrate solution containing 20 mM pNPP (Acros Organics BVBA). The absorbance increase in samples was measured at 405 nm using a Tecan Infinite M1000 multiplate reader (Tecan AG) for 30 min at 37 °C.

### NanoLuc (nLuc) measurement

NanoLuc concentrations in cell culture supernatants were quantified with the Nano-Glo Luciferase Assay System (N1110, Promega). 10 μL of cell culture supernatant was mixed with 10 μL of buffer/substrate mix (50:1) in 384-well plates (781076, Greiner Bio-One), which were briefly centrifuged at 1200 rpm and incubated for 5 min at room temperature. Luminescence intensity was measured using a Tecan Spark multiplate reader (Tecan AG).

### Guide RNA design

Guide sequences targeting *VEGFA*, *TNFRSF1A* and *ACE2* were designed with the online tool (http://guides.sanjanalab.org/#/). Three gRNAs with high ON-target efficiency and low OFF-target specificity for each target were selected.

### Sample preparation for NGS (deep sequencing quantification of Cas9-induced indels)

To quantify the efficiency of gene editing, genomic DNA of either 200,000 cells (experiments performed in 24-well plates) was isolated using a Monarch Genomic DNA Purification Kit (T3010S, New England BioLabs) or 40,000 cells (experiments performed in 96-well plates) was isolated by resuspending cells into quick extraction buffer (QE, in mM: 1 CaCl_2_, 3 MgCl_2_, 1 EDTA, 10 Tris pH 7.5; 1% Triton X-100, and 0.2 mg/mL proteinase K freshly added before use) followed by incubation at 65 °C for 10 min, 68 °C for 10 min, and 98 °C for 10 min. Genomic DNA was used as a template for a first PCR to amplify ~150 bp around the Cas9 cut site with gene-specific primers containing adapters (Supplementary Table S7) (0.5 µM, fwd: ACACTCTTTCCCTACACGACGCTCTTCCGATCT + forward gene-specific sequence; rev: GTGACTGGAGTTCAGACGTGTGCTCTTCCGATC + reverse gene-specific sequence). PCR 1 was performed with KAPA HiFi Ready Mix as follows: (1) 95 °C for 3 min; (2) 98 °C for 20 s, 65 °C for 15 s, 72 °C for 20 s (15 cycles); (3) 72 °C for 2 min. PCR 2 to index samples with P5 and P7 primers (0.25 µM, P5: AATGATACGGCGACCACCGAGATCTACAC NNNNNNNN ACACTCTTTCCCTACACGACGCTCTTCCGATCT; P7: CAAGCAGAAGACGGCATACGAGAT- NNNNNNNN GTGACTGGAGTTCAGACGTGTGCTCTTCCGATC) was performed as follows: (1) 95 °C for 3 min; (2) 98 °C for 20 s, 70 °C for 15 s, 72 °C 20 sec (15 cycles); (3) 72 °C for 2 min. Samples were purified with a PCR purification & concentration kit (D4013, Zymo Research), and run on a 2% E-Gel (G402022, Thermo Fisher Scientific). The PCR product (~ 250 bp) was extracted from the gel with a QIAquick Gel Extraction Kit (28706×4, QIAGEN). Deep sequencing was done with a NextSeq 550 150 cycle kit with the following cycle distribution: 150 to read 1, 8 to index 1, and 8 to index 2.

### Indel analysis

Deep sequencing libraries for indel analysis were analyzed with CRISPresso2^51^ with the following parameters: -r1 “fastq file name”; -a “amplicon sequence”; -c “amplicon sequence”; -g “gRNA sequence”; --default_min_aln_score 60; --plot_window_size 20; --min_bp_quality_or_N 0; --exclude_bp_from_left 15; --exclude_bp_from_right 15; -w 1; and -wc -3.

### IL-12 quantification

IL-12 in the cell supernatant was quantified with a human IL-12(p40) ELISA Kit (KAC1561, Thermo Fisher Scientific) according to the manual provided by the manufacturer.

### Animal studies

Eight-week-old male BALB/c or C57BL/6 mice (Charles River Laboratory, Lyon, France), weighing 20–25 g, were used. The animals were housed in a controlled room set at 22 °C, 50% humidity, 12-h light-dark cycle with ad-libitum access to standard diet and drinking water. Animals were randomly assigned to experimental groups. After collection of blood samples, serum was isolated for analysis using BD Microtainer SST tubes (365967, Becton Dickinson) according to the manufacturer’s instruction (30 min incubation in the dark followed by 5 min centrifugation at 8000× *g*). All experiments involving animals were performed according to the directives of the European Community Council (2010/63/EU), approved by the French Republic (project no. DR2018-40v5 and APAFIS #16753), and carried out by Shuai Xue and Ghislaine Charpin-El Hamri (no. 69266309) at the University of Lyon, Institut Universitaire de Technologie (IUT), F69622 Villeurbanne Cedex, France.

### Hydrodynamic plasmid delivery

Mice were injected into the tail vein with 2 mL of saline, containing plasmids coding for either iCROP-fLuc, iCROP-sr-IκBα or a combination of iCROP-Cas9 and sgRNA(mPCSK9) 24 h before starting the inducer treatment.

### Inducer preparation for in vivo application and administration

Rupintrivir was formulated as a 1.2 mg/mL solution (10% DMSO, 40% PEG400 and 50% saline) and mice were induced by intraperitoneally injecting 100 µL of this solution.

### In vivo bioluminescence

Mice engineered to express iCROP-fLuc were first sedated with isoflurane and intraperitoneally injected with 250 μL of D-luciferin (LUCK-100, Gold Biotechnology) at a concentration of 15 mg/mL (dissolved in PBS), 5 min before measurement of the luminescence intensity with an IVIS Imaging System (PerkinElmer).

### Cytokine quantification

TNFα in mouse serum was quantified with a TNF alpha Mouse ELISA Kit (BMS607-3, Thermo Fisher Scientific). IL-1β in mouse serum was quantified with a Mouse IL-1β ELISA Kit (RAB0274-1KT, Sigma-Aldrich). IL-6 in mouse serum was quantified with an IL-6 Mouse ELISA Kit (BMS603-2, Thermo Fisher Scientific). All assays were performed according to the instructions provided by the manufacturer.

### Quantification of PCSK9

PCSK9 in mouse serum was quantified with a Mouse Proprotein Convertase 9/PCSK9 Quantikine ELISA Kit (MPC900, R&D Systems) according to the manufacturer’s instructions.

### LDL-cholesterol measurement

LDL-Cholesterol levels in mouse serum were quantified with a Cholesterol assay kit (ab65390, Abcam) according to the instructions provided by the manufacturer.

### Data analysis and visualization

Statistical evaluation was conducted by using Welch’s two-tailed *t*-test to compare two sets of data as implemented in Prism GraphPad 9 (GraphPad Software Inc., San Diego, CA). Figures were created by using Adobe Illustrator (Adobe Inc.).

### Supplementary information

Supplementary Information
