# Supplementary material for: Integrated compact regulators of protein activity enable control of signaling pathways and genome-editing in vivo
Source: Cell Discov. 2024 Jan 23;10:9. doi: 10.1038/s41421-023-00632-1 (PMC10805712; doi:10.1038/s41421-023-00632-1)
Supplement: Supplementary file 1 — Supplementary Information [file 41421_2023_632_MOESM1_ESM.pdf]

Supplementary Materials

Integrated compact regulators of protein activity enable control of signaling pathways and genome-editing *in vivo*

Nik Franko[1], António José da Silva Santinha[1], Shuai Xue[1], Haijie Zhao[1], Ghislaine Charpin-El Hamri[2], Randall Jeffrey Platt[1], Ana Palma Teixeira[1], Martin Fussenegger[1,3]

[1]Department of Biosystems Science and Engineering, ETH Zurich, Klingelbergstrasse 48, CH-4056 Basel, Switzerland.

[2]Département Génie Biologique, Institut Universitaire de Technologie, Université Claude Bernard Lyon 1, F-69622, Villeurbanne, Cedex, France.

[3]Faculty of Science, University of Basel, Klingelbergstrasse 48, CH-4056 Basel, Switzerland.

[*]Correspondence: martin.fussenegger@bsse.ethz.ch

Contents

1. **Supplementary Tables S1-S7**
2. **Supplementary Figures S1-S16**

Supplementary Table S1. Protease sequences

Protease	Amino acid sequence
**HIVp**	MPQITLWQRPLVTIKIGGQLKEALLDTGADDTVLEEMNLPGRWKPKMIGGIGGFI KVRQYDQILIEICGHKAIGTVLVGPTPVNIIGRNLLTQIGCTLNF
**HCVp**	MSGTGCVVIVGRIVLSGSGTSAPITAYAQQTRGLLGCIITSLTGRDKNQVEGEVQIV STATQTFLATCINGVCWAVYHGAGTRTIASPKGPVIQMYTNVDQDLVGWPAPQG SRSLTPCTCGSSDLYLVTRHADVIPVRRRGDSRGSLLSPRPISYLKGSSGGPLLCPA GHAVGLFRAAVCTRGVAKAVDFIPVENLETTMRSPVFTDNSSPPAVTLTHPITKID REVLYQEF
**HRVp**	MGPNTEFALSLLRKNIMTITTSKGEFTGLGIHDRVCVIPTHAQPGDDVLVNGQKIR VKDKYKLVDPENINLELTVLTLDRNEKFRDIRGFISEDLEGVDATLVVHSNNFTNT ILEVGPVTMAGLINLSSTPTNRMIRYDYATKTGQCGGVLCATGKIFGIHVGGNGR QGFSAQLKKQYFVEKQ

Supplementary Table S2. Protease cleavage sites

Cleavage site	Amino acid sequence
**HIVp CS**	SQVSQNYPIVQNLQ
**HCVp CS**	EDVVPCSMGS
**HRVp CS**	LEVLFQGP

Supplementary Table S3. Plasmids used and designed in this study.

pTS1106	P_{hCMV}-TetR-pA<br><br>Mammalian expression plasmid encoding P_{hCMV}-driven DNA binding domain TetR.	[1]
pTS1017	O_{TetR}_$P_{hCMVmin}$-SEAP-pA<br><br>Mammalian reporter plasmid encoding O_{TetR}-$P_{hCMVmin}$ driven expression of SEAP reporter protein.	[1]
pDA326	P_{TREBI}-Citrine-2A-SEAP-sTRSV-pA<br><br>Mammalian reporter plasmid encoding for P_{TREBI}-driven Citrine-2A-SEAP expression cassette with a sTRSV ribozyme-dependent destabilization module in the 3'-UTR.	[2]
BB3-P_{mPGK1}	P_{mPGK1}-MCS-pA<br><br>Mammalian expression plasmid encoding P_{mPGK1} and multi cloning site (MCS).	[1]
BB3-fLuc	P_{hCMV}-fLuc-pA<br><br>Mammalian expression plasmid encoding P_{hCMV}- driven firefly luciferase (fLuc).	[1]
pcDNA3.1(+)	P_{hCMV}-MCS-pA<br><br>Cloning vector for P_{hCMV} driven constitutive expression of target genes with MCS.	ThermoFisher
pDF145	P_{T7}-SpAH-Env140ac<br><br>In vitro RNA production plasmid without mammalian promoter activity.	[3]

rTetR-NS3-VP64-p65_IRES_mCherry	Plasmid encoding HCV protease.	Addgene plasmid #112628
pCDNA3/GFP-PR	Plasmid encoding HIV protease.	Addgene plasmid #20253
pBabe-Puro-IKBalpha-mut (super repressor)	Plasmid encoding super repressor IκBα (sr-IκBα).	Addgene plasmid #15291
pMIG-hNFATc2/C – AVITEV	Plasmid encoding NFAT1.	Addgene plasmid #74058
NFATC1	Plasmid encoding NFAT2.	DNASU HsCD00436665
pRSV-p65	Plasmid encoding RelA (p65).	Addgene plasmid #106453
MYD88	Plasmid encoding MyD88.	DNASU HsCD00002377
pYL43	$P_{NF\text{-}\kappa B}$-SEAP-pA<br><br>$P_{NF\text{-}\kappa B}$-driven SEAP reporter protein expression vector.	[4]
pMX57	P_{3xNFAT}-SEAP-pA<br><br>P_{3xNFAT}-driven SEAP reporter protein expression vector.	[5]
pKK89	P_{3xNFAT}-ss-nLuc-Fc-pA<br><br>P_{3xNFAT}-driven stabilized secreted nLuc reporter protein expression vector.	[6]
pGM74	P_{hCMV}-dCas9-NLS-pA<br><br>P_{hCMV} driven *Streptococcus pyogenes* catalytically dead CRISPR associated protein 9 (dCas9) expression vector.	[7]
pKK44	P_{hCMV}-MCP-$p65_{TA}$-$HSF1_{TA}$-pA<br><br>P_{hCMV}-driven expression of a fusion protein that consists of MCP, the transactivation domain of p65 ($p65_{TA}$) and the transactivation domain of heat shock factor 1 ($HSF1_{TA}$).	[7]
pSP20	P_{hINS}-SEAP-pA<br><br>P_{hINS}-driven SEAP reporter protein expression vector.	[7]
pGM70	P_{hU6}-$sgRNA_{hINS}$<br><br>P_{hU6}-driven sgRNA complementary to a sequence in the human insulin promoter.	[7]
pKK158	P_{hU6}-$sgRNA_{IL\text{-}12B}$<br><br>P_{hU6}-driven sgRNA complementary to a sequence in the human IL-12B promoter.	[7]
psgRNA(MS2)	sgRNA cloning vector with MCP-binding loops at tetraloop and stemloop 2.	Addgene plasmid #61424
pFS220	P_{Ca2}-Citrine-pA<br><br>Mammalian calcium-dependent citrine expression vector.	[8]
pTS395	PhCMV-SB100-pA<br><br>P_{hCMV}-driven Sleeping Beauty transposase mammalian expression vector.	[1]
pTS1024	P_{RPBSA}-YPet-p2a-PuroR-pA<br><br>Vector encoding P_{RPBSA}-driven YPet in combination with a marker for puromycin selection (PuroR).	[1]
pTS1107	Vector for stable integration of up to three cassettes via Sleeping Beauty transposase (5’ITR-A1-pA::A2-pA::A3-pA-3’ITR)	[1]

pNF151	$O_{TetR}\_P_{hCMVmin}$-nLuc-sTRSV-pA<br><br>$O_{TetR}\_P_{hCMVmin}$- driven expression of nLuc reporter protein with a sTRSV ribozyme-dependent destabilization module in the 3'-UTR.	[9]
pNF167	P_{mPGK1}-Mpro(S2)-15gs-TetR-$_{Mpro}CS_{OPT}$-NLS-VP64-pA<br><br>Mammalian expression plasmid encoding P_{mPGK1}-driven SARS-CoV-2 Mpro linked to N-terminal of DNA binding domain TetR fused to transcription activation domain VP64 with fusion linker containing $Mpro_{OPT}$ cleavage site and NLS.	[9]
pNF212	P_{mPGK1}-HIVp-TetR-CS-VP64-pA<br><br>Mammalian expression plasmid encoding P_{mPGK1}-driven HIVp linked to N-terminal of DNA binding domain TetR fused to transcription activation domain VP64 with fusion linker containing HIVp cleavage site and NLS.<br><br>HIVp was amplified from Addgene plasmid #20253 using oNF376 and oNF377. TetR was amplified from pTS1106 using oNF260 and oNF378. PCR assembly reaction using oNF376 and oNF378 was used to fuse HIVp and TetR-$_{HIVp}$CS with 15gs linker. The PCR fragment was digested with EcoRI/BamHI and ligated into pNF167 (EcoRI/BamHI).	This work
pNF214	P_{mPGK1}-TetR-HIVp-VP64-pA<br><br>Mammalian expression plasmid encoding P_{mPGK1}-driven HIVp with its cleavage sites on C-end linked to C-terminal of DNA binding domain TetR fused to transcription activation domain VP64 with fusion linker containing NLS.<br><br>TetR was amplified from pTS1106 using oNF249 and oNF379. HIVp was amplified in a two-step process from Addgene plasmid #20253, first using oNF380 and oNF377 and second, using oNF380 and oNF358. PCR assembly reaction using oNF249 and oNF358 was used to fuse TetR and HIVp. The PCR fragment was digested with EcoRI/BamHI and ligated into pNF167 (EcoRI/BamHI).	This work
pNF217	P_{mPGK1}-sr-IκBα$_{CS\text{-}L131}$-pA<br><br>Mammalian expression plasmid encoding P_{mPGK1}-driven sr-IκBα containing HRVp CS located upstream of L131.<br><br>sr-IκBα was amplified from Addgene plasmid #15291, first with oNF383 and oNF384 and second with oNF385 and oNF390 to introduce CS, followed by PCR assembly reaction using oNF383 and oNF390. The PCR fragment was digested with EcoRI/XbaI and ligated into pNF167 (EcoRI/XbaI).	This work
pNF218	P_{mPGK1}-sr-IκBα$_{CS\text{-}P170}$-pA<br><br>Mammalian expression plasmid encoding P_{mPGK1}-driven sr-IκBα containing HRVp CS located upstream of P170.<br><br>sr-IκBα was amplified from Addgene plasmid #15291, first with oNF383 and oNF386 and second with oNF387 and oNF390 to introduce CS, followed by PCR assembly reaction using oNF383 and oNF390. The PCR fragment was digested with EcoRI/XbaI and ligated into pNF167 (EcoRI/XbaI).	This work
pNF219	P_{mPGK1}-sr-IκBα$_{CS\text{-}G206}$-pA<br><br>Mammalian expression plasmid encoding P_{mPGK1}-driven sr-IκBα containing HRVp CS located upstream of G206.	This work

	sr-IκBα was amplified from Addgene plasmid #15291, first with oNF383 and oNF388 and second with oNF389 and oNF390 to introduce CS, followed by PCR assembly reaction using oNF383 and oNF390. The PCR fragment was digested with EcoRI/XbaI and ligated into pNF167 (EcoRI/XbaI).	
pNF220	P_{mPGK1}-sr-IκBα-pA<br><br>Mammalian expression plasmid encoding P_{mPGK1}-driven sr-IκBα.<br><br>sr-IκBα was amplified from Addgene plasmid #15291 using oNF383 and oNF390. The PCR fragment was digested with EcoRI/XbaI and ligated into pNF167 (EcoRI/XbaI).	This work
pNF233	P_{mPGK1}-HRVp$_{\Delta Q182}$-TetR-VP64-pA<br><br>Mammalian expression plasmid encoding P_{mPGK1}-driven TetR-VP64 with N-terminally fused HRVp$_{\Delta Q182}$.<br><br>HRVp$_{\Delta Q182}$ was amplified from Twist gene fragment DNA_twist_HRVp using oNF353 and oNF403. TetR was amplified from pTS1106 using oNF260 and oNF261. PCR assembly reaction using oNF353 and oNF261 was used to fuse HRVp$_{\Delta Q182}$ and TetR with 15gs linker. The PCR fragment was digested with EcoRI/BamHI and ligated into pNF167 (EcoRI/BamHI).	This work
pNF234	P_{mPGK1}-HRVp$_{\Delta Q182}$-sr-IκBα$_{CS\text{-}P170}$-pA<br><br>Mammalian expression plasmid encoding P_{mPGK1}-driven sr-IκBα$_{CS\text{-}P170}$ with N-terminally fused HRVp$_{\Delta Q182}$.<br><br>HRVp$_{\Delta Q182}$ was amplified from Twist gene fragment DNA_twist_HRVp, first using oNF353 and oNF403, followed by second amplification using oNF353 and oNF358. The PCR fragment was digested with EcoRI/BamHI and ligated into pNF218 (EcoRI/BamHI).	This work
pNF244	P_{mPGK1}-HRVp$_{\Delta Q182}$-TetR-CS-VP64-pA<br><br>Mammalian expression plasmid encoding P_{mPGK1}-driven HRVp$_{\Delta Q182}$ linked to N-terminal of DNA binding domain TetR fused to transcription activation domain VP64 with fusion linker containing HRVp CS and NLS.<br><br>HRVp was amplified from Twist gene fragment DNA_twist_HRVp using oNF353 and oNF403. TetR was amplified from pTS1106 using oNF260 and oNF352. PCR assembly reaction using oNF353 and oNF352 was used to fuse HRVp$_{\Delta Q182}$ and TetR-CS with 15gs linker. The PCR fragment was digested with EcoRI/BamHI and ligated into pNF167 (EcoRI/BamHI).	This work
pNF247	P_{mPGK1}-HRVp$_{\Delta Q182}$-fLuc$_{CS\text{-}K491}$-pA<br><br>Mammalian expression plasmid encoding P_{mPGK1}-driven HRVp$_{\Delta Q182}$ linked to N-terminal of fLuc containing HRVp CS.<br><br>HRVp$_{\Delta Q182}$ was amplified from pNF244 using oNF353 and oNF358 and digested with EcoRI/BamHI. HRVp CS was introduced into fLuc by amplifying BB3-fLuc first with oNF374 and oNF410 and second with oNF409 and oNF375 followed by PCR assembly reaction using oNF374 and oNF375. The PCR fragment was digested with BamHI/XbaI. Both fragments were ligated into pNF167 (EcoRI/XbaI).	This work

pNF249	P_{mPGK1}-MCP-HRVp$_{\Delta Q182}$-CS-VP64-pA<br><br>Mammalian expression plasmid encoding P_{mPGK1}-driven MCP N-terminally fused to HRVp$_{\Delta Q182}$ with CS and VP64.<br><br>MCP was amplified from pKK44 using oNF237 and oNF415. HRVp$_{\Delta Q182}$-CS was amplified from pNF244 using oNF414 and oNF357. PCR assembly reaction using oNF237 and oNF357 was used to construct MCP-HRVp$_{\Delta Q182}$-CS. The PCR fragment was digested with EcoRI/BamHI and ligated into pNF167 (EcoRI/BamHI).	This work
pNF251	P_{mPGK1}-HCVp-TetR-CS-VP64-pA<br><br>Mammalian expression plasmid encoding P_{mPGK1}-driven hepatitis C virus protease linked to N-terminal of DNA binding domain TetR fused to transcription activation domain VP64 with fusion linker containing HCVp cleavage site and NLS.<br><br>HCVp was amplified from Addgene plasmid #112628 using oNF468 and oNF469. TetR was amplified from pTS1106 using oNF260 and oNF471. PCR assembly reaction using oNF468 and oNF471 was used to fuse HCVp and TetR-CS with 15gs linker. The PCR fragment was digested with EcoRI/BamHI and ligated into pNF167 (EcoRI/BamHI).	This work
pNF255	P_{mPGK1}-HRVp$_{\Delta Q182}$-dCas9$_{CS\text{-}V713}$-pA<br><br>Mammalian expression plasmid encoding P_{mPGK1}-driven HRVp$_{\Delta Q182}$ fused to dCas9 containing HRVp CS after V713.<br><br>CS was inserted by amplifying pGM74 with oNF464/oNF465 and oNF466/oNF467, followed by PCR assembly reaction using oNF464 and oNF467. The PCR fragment was digested with BamHI/XbaI and ligated into pNF234 (BamHI/XbaI).	This work
pNF263	P_{mPGK1}-HRVp$_{\Delta Q182}$-pA<br><br>Mammalian expression plasmid encoding P_{mPGK1}-driven HRVp$_{\Delta Q182}$.<br><br>HRVp$_{\Delta Q182}$ was amplified from pNF247 using oNF353 and oNF484. The PCR fragment was digested with EcoRI/XbaI and ligated into oNF167(EcoRI/XbaI).	This work
pNF265	P_{mPGK1}-fLuc$_{CS\text{-}K491}$-pA<br><br>Mammalian expression plasmid encoding P_{mPGK1}-driven fLuc$_{CS\text{-}K491}$.<br><br>fLuc$_{CS\text{-}K491}$ was digested from pNF247 using BamHI/XbaI and ligated into pNF218 (BamHI/XbaI).	This work
pNF286	P_{mPGK1}-Cas9-pA<br><br>Mammalian expression plasmid encoding P_{mPGK1}-driven Cas9.<br><br>Cas9 was amplified from pGM74 using three sets of primers (oNF495/497), (oNF496/499) and (oNF498/467) to mutate dCas9 into Cas9 and remove internally occurring MluI site, followed by a series of PCR assembly reactions. The PCR fragment was digested with BamHI/XbaI and ligated into pNF218 (BamHI/XbaI).	This work
pNF287	P_{mPGK1}-NFAT1$_{S172\text{-}G701}$-HRVp$_{\Delta Q182}$-CS-VP64-pA	This work

	Mammalian expression plasmid encoding P_{mPGK1}-driven $NFAT1_{S172\text{-}G701}$ N-terminally fused to $HRVp_{\Delta Q182}$ with CS and VP64.<br><br>$NFAT1_{S172\text{-}G701}$ was amplified from Addgene plasmid #74058 with three sets of primers (oNF503/oNF505), (oNF504/oNF507) and (oNF506/oNF508) to remove internal BamHI sites, followed by PCR assembly reactions. The full fragment was amplified using oNF503 and oNF508. $HRVp_{\Delta Q182}$-CS was amplified from pNF244 with oNF408 and oNF357. PCR assembly reaction using oNF503 and oNF357 afforded $NFAT1_{S172\text{-}G701}$-$HRVp_{\Delta Q182}$-CS, which was digested with EcoRI/BamHI and ligated into pNF167 (EcoRI/BamHI).	
pNF288	P_{mPGK1}-$NFAT2_{P81\text{-}Q593}$-$HRVp_{\Delta Q182}$-CS-VP64-pA<br><br>Mammalian expression plasmid encoding P_{mPGK1}-driven $NFAT2_{P81\text{-}Q593}$ N-terminally fused to $HRVp_{\Delta Q182}$ with CS and VP64.<br><br>$NFAT2_{P81\text{-}Q593}$ was amplified from plasmid NFATC1 using oNF509 and oNF510. $HRVp_{\Delta Q182}$-CS was amplified from pNF244 with oNF408 and oNF357. PCR assembly reaction using oNF509 and oNF357 afforded $NFAT2_{P81\text{-}Q593}$-$HRVp_{\Delta Q182}$-CS, which was digested with EcoRI/BamHI and ligated into pNF167 (EcoRI/BamHI).	This work
pNF293	O_{TetR}-$P_{hCMVmin}$-SEAP-sTRSV-pA<br><br>Mammalian reporter plasmid encoding for O_{TetR}-$P_{hCMVmin}$-driven expression of SEAP reporter protein with a sTRSV ribozyme-dependent destabilization module in the 3'-UTR.<br><br>sTRSV was excised from pDA326 using XbaI/SpeI and ligated into pTS1017 (XbaI).	This work
pNF298	P_{mPGK1}-$HRVp_{\Delta Q182}$-$dCas9_{CS\text{-}E311}$-pA<br><br>Mammalian expression plasmid encoding P_{mPGK1}-driven $HRVp_{\Delta Q182}$ fused to dCas9 containing HRVp CS after E311.<br><br>CS was inserted by amplifying pGM74 with oNF464/oNF531 and oNF530/oNF467, followed by PCR assembly reaction using oNF464 and oNF467. The PCR fragment was digested with BamHI/XbaI and ligated into pNF234 (BamHI/XbaI).	This work
pNF299	P_{mPGK1}-$HRVp_{\Delta Q182}$-dCas9-CS_{R535}-pA<br><br>Mammalian expression plasmid encoding P_{mPGK1}-driven $HRVp_{\Delta Q182}$ fused to dCas9 containing HRVp CS after R535.<br><br>CS was inserted by amplifying pGM74 with oNF464/oNF533 and oNF532 and oNF467, followed by PCR assembly reaction using oNF464 and oNF467. The PCR fragment was digested with BamHI/XbaI and ligated into pNF234 (BamHI/XbaI).	This work
pNF302	P_{mPGK1}-TetR-$HRVp_{\Delta Q182}$-CS-VP64-pA<br><br>Mammalian expression plasmid encoding P_{mPGK1}-driven $HRVp_{\Delta Q182}$ with its cleavage sites on C-end linked to C-terminal of DNA binding domain TetR fused to transcription activation domain VP64 with fusion linker containing NLS.<br><br>TetR was amplified from pTS1106 using oNF249 and oNF379. $HRVp_{\Delta Q182}$-CS was amplified from pNF244 using oNF355 and oNF357. PCR assembly reaction using oNF249 and oNF357 was used to fuse TetR and $HRVp_{\Delta Q182}$-CS. The PCR fragment was	This work

	digested with EcoRI/BamHI and ligated into pNF167 (EcoRI/BamHI).	
pNF303	P_{mPGK1}-TetR-HRVp-VP64<br><br>Mammalian expression plasmid encoding P_{mPGK1}-driven HRVp linked to C-terminal of DNA binding domain TetR fused to transcription activation domain VP64 with fusion linker containing NLS.<br><br>TetR was amplified from pTS1106 using oNF249 and oNF379. HRVp was amplified in a two-step process from pNF244, first using oNF355 and oNF354 and second using oNF355 and oNF358. PCR assembly reaction using oNF249 and oNF358 was used to fuse TetR and HRVp. The PCR fragment was digested with EcoRI/BamHI and ligated into pNF167 (EcoRI/BamHI).	This work
pNF304	P_{mPGK1}-TetR-CS-HCVp-CS-VP64<br><br>Mammalian expression plasmid encoding P_{mPGK1}-driven HCVp with its cleavage sites on N- and C-end linked to C-terminal of DNA binding domain TetR fused to transcription activation domain VP64 with fusion linker containing NLS.<br><br>TetR was amplified from pTS1106 using oNF249 and oNF379. HCVp was amplified from Addgene plasmid #112628 using oNF477 and oNF478. PCR assembly reaction using oNF249 and oNF478 was used to fuse TetR and CS-HCVp-CS. The PCR fragment was digested with EcoRI/BamHI and ligated into pNF167 (EcoRI/BamHI).	This work
pNF305	P_{mPGK1}-TetR-HRVp$_{\Delta Q182}$-VP64<br><br>Mammalian expression plasmid encoding P_{mPGK1}-driven HRVp$_{\Delta Q182}$ linked to C-terminal of DNA binding domain TetR fused to transcription activation domain VP64 with fusion linker containing NLS.<br><br>TetR was amplified from pTS1106 using oNF249 and oNF379. HRVp$_{\Delta Q182}$ was amplified from pNF244 using oNF355 and oNF358. PCR assembly reaction using oNF249 and oNF358 was used to fuse TetR and HRVp$_{\Delta Q182}$. The PCR fragment was digested with EcoRI/BamHI and ligated into pNF167 (EcoRI/BamHI).	This work
pNF310	P_{mPGK1}-MCP-HRVp-VP64-pA<br><br>Mammalian expression plasmid encoding P_{mPGK1}-driven MCP N-terminally fused to HRVp and VP64.<br><br>MCP was amplified from pKK44 using oNF237 and oNF415. HRVp was amplified from pNF303 using oNF414 and oNF358. PCR assembly reaction using oNF237 and oNF358 was used to construct MCP-HRVp. The PCR fragment was digested with EcoRI/BamHI and ligated into pNF167 (EcoRI/BamHI).	This work
pNF311	P_{mPGK1}-NFAT1$_{S172\text{-}G701}$-HRVp-VP64-pA<br><br>Mammalian expression plasmid encoding P_{mPGK1}-driven NFAT1$_{S172\text{-}G701}$ N-terminally fused to HRVp and VP64.<br><br>NFAT1$_{S172\text{-}G701}$ was amplified from pNF287 using oNF503 and oNF508. HRVp was amplified from pNF303 with oNF408 and oNF358. PCR assembly reaction using oNF503 and oNF358 obtained NFAT1$_{S172\text{-}G701}$-HRVp which was digested with EcoRI/BamHI and ligated into pNF167 (EcoRI/BamHI).	This work
pNF313	P_{mPGK1}-NFAT1$_{S172\text{-}G701}$-HRVp-NFAT1$_{L702\text{-}T925}$-pA	This work

	Mammalian expression plasmid encoding P_{mPGK1}-driven NFAT1, modified to contain HRVp between G701 and L702.<br><br>$NFAT1_{L702-T925}$ was amplified from Addgene plasmid #74058 using oNF539 and oNF540. The PCR fragment was digested with BamHI/XbaI and ligated into pNF311 (BamHI/XbaI).	
pNF316	P_{mPGK1}-N-fLuc-HRVp-C-fLuc-pA<br><br>Mammalian expression plasmid encoding P_{mPGK1}-driven fLuc, modified to contain HRVp between K491 and T492.<br><br>N-terminal part of fLuc was amplified from BB3-fLuc using oNF374 and oNF534. HRVp was amplified from pNF303 using oNF408 and oNF354. C-terminal part of fLuc was amplified from BB3-fLuc using oNF545 and oNF375. HRVp and C-fLuc were fused by PCR assembly using oNF408 and oNF375, followed by another PCR assembly to add N-fLuc using oNF374 and oNF375. The PCR fragment was digested with BamHI/XbaI and ligated into pNF220 (BamHI/XbaI).	This work
pNF318	P_{hCMV}_P_{T7}-$HRVp_{\Delta Q182}$-$fLuc_{CS-K491}$-pA<br><br>Mammalian expression plasmid encoding P_{hCMV}_P_{T7}-driven $HRVp_{\Delta Q182}$ linked to N-terminal of fLuc containing HRVp CS.<br><br>P_{hCMV}_P_{T7} was digested from pcDNA3.1(+) using MluI/EcoRI and ligated into pNF247 (MluI/EcoRI).	This work
pNF322	P_{hU6}-$sgRNA_{EMX1}$<br><br>P_{hU6}-driven sgRNA complementary to a sequence in the human *EMX1* gene.<br><br>Oligonucleotides oNF593 and oNF594 were annealed and ligated into BbsI-digested psgRNA(MS2).	This work
pNF325	P_{mPGK1}-$HRVp_{\Delta Q182}$-$Cas9_{CS-R535}$-pA<br><br>Mammalian expression plasmid encoding P_{mPGK1}-driven $HRVp_{\Delta Q182}$ fused to Cas9 containing HRVp CS after R535.<br><br>CS was inserted by amplifying pNF286 with oNF464/oNF533 and oNF532/oNF467, followed by PCR assembly reaction using oNF464 and oNF467. The PCR fragment was digested with BamHI/XbaI and ligated into pNF234 (BamHI/XbaI).	This work
pNF334	P_{mPGK1}-dCas9-pA<br><br>Mammalian expression plasmid encoding P_{mPGK1}-driven dCas9.<br><br>dCas9 was amplified from pGM74 using oNF464 and oNF467. The PCR fragment was digested with BamHI/XbaI and ligated into pNF218 (BamHI/XbaI).	This work
pNF335	P_{mPGK1}-$dCas9_{CS-E311}$-pA<br><br>Mammalian expression plasmid encoding P_{mPGK1}-driven dCas9 containing HRVp CS after E311.<br><br>CS was inserted by amplifying pNF334 with oNF464/oNF531 and oNF530 and oNF467, followed by PCR assembly reaction using oNF464 and oNF467. The PCR fragment was digested with BamHI/XbaI and ligated into pNF218 (BamHI/XbaI).	This work
pNF336	P_{mPGK1}-$dCas9_{CS-R535}$-pA<br><br>Mammalian expression plasmid encoding P_{mPGK1}-driven dCas9 containing HRVp CS after R535.	This work

	CS was inserted by amplifying pNF334 with oNF464/oNF533 and oNF532 and oNF467, followed by PCR assembly reaction using oNF464 and oNF467. The PCR fragment was digested with BamHI/XbaI and ligated into pNF218 (BamHI/XbaI).	
pNF337	P_{mPGK1}-dCas9$_{CS\text{-}V713}$-pA<br><br>Mammalian expression plasmid encoding P_{mPGK1}-driven dCas9 containing HRVp CS after V713.<br><br>CS was inserted by amplifying pNF334 with oNF464/oNF465 and oNF466 and oNF467, followed by PCR assembly reaction using oNF464 and oNF467. The PCR fragment was digested with BamHI/XbaI and ligated into pNF218 (BamHI/XbaI).	This work
pNF340	P_{mPGK1}-HRVp$_{\Delta Q182}$-fLuc-pA<br><br>Mammalian expression plasmid encoding P_{mPGK1}-driven fLuc with N-terminally fused HRVp$_{\Delta Q182}$.<br><br>HRVp$_{\Delta Q182}$ was amplified from pNF244 using oNF353 and oNF358 and digested with EcoRI/BamHI. FLuc was amplified from bb3-fLuc using oNF374 and oNF375 and digested with BamHI/XbaI. Both fragments were ligated into pNF167 (EcoRI/XbaI).	This work
pNF347	P_{mPGK1}-NFAT1-pA<br><br>Mammalian expression plasmid encoding P_{mPGK1}-driven full length NFAT1.<br><br>NFAT1 was amplified from Addgene plasmid #74058 with oNF601 and oNF540. The PCR fragment was digested with EcoRI/XbaI and ligated into pNF167 (EcoRI/XbaI).	This work
pNF348	P_{mPGK1}-NFAT1$_{S172\text{-}T925}$-pA<br><br>Mammalian expression plasmid encoding P_{mPGK1}-driven shortened NFAT1 spanning from S172 to T925.<br><br>NFAT1$_{S172\text{-}T925}$ was amplified from Addgene plasmid #74058 with oNF503 and oNF540. The PCR fragment was digested with EcoRI/XbaI and ligated into pNF167 (EcoRI/XbaI).	This work
pNF359	P_{hU6}-sgRNA$_{VEGFA\text{-}1}$<br><br>P_{hU6}-driven sgRNA complementary to a sequence in the exon 4 of human *VEGFA* gene.<br><br>Oligonucleotides oNF638 and oNF639 were annealed and ligated into BbsI-digested psgRNA(MS2).	This work
pNF360	P_{hU6}-sgRNA$_{VEGFA\text{-}2}$<br><br>P_{hU6}-driven sgRNA complementary to a sequence in the exon 5 of human *VEGFA* gene.<br><br>Oligonucleotides oNF640 and oNF641 were annealed and ligated into BbsI-digested psgRNA(MS2).	This work
pNF361	P_{hU6}-sgRNA$_{VEGFA\text{-}3}$<br><br>P_{hU6}-driven sgRNA complementary to a sequence in the exon 8 of human *VEGFA* gene.<br><br>Oligonucleotides oNF642 and oNF643 were annealed and ligated into BbsI-digested psgRNA(MS2).	This work
pNF362	P_{hU6}-sgRNA$_{TNFRSF1A\text{-}1}$	This work

	P_{hU6}-driven sgRNA complementary to a sequence in the exon 8 of human *TNFRSF1A* gene.<br><br>Oligonucleotides oNF644 and oNF645 were annealed and ligated into BbsI-digested psgRNA(MS2).	
pNF363	P_{hU6}-sgRNA$_{TNFRSF1A\text{-}2}$<br><br>P_{hU6}-driven sgRNA complementary to a sequence in the exon 8 of human *TNFRSF1A* gene.<br><br>Oligonucleotides oNF646 and oNF647 were annealed and ligated into BbsI-digested psgRNA(MS2).	This work
pNF364	P_{hU6}-sgRNA$_{TNFRSF1A\text{-}3}$<br><br>P_{hU6}-driven sgRNA complementary to a sequence in the exon 8 of human *TNFRSF1A* gene.<br><br>Oligonucleotides oNF648 and oNF649 were annealed and ligated into BbsI-digested psgRNA(MS2).	This work
pNF365	P_{hU6}-sgRNA$_{ACE2\text{-}1}$<br><br>P_{hU6}-driven sgRNA complementary to a sequence in the exon 18 of human *ACE2* gene.<br><br>Oligonucleotides oNF650 and oNF651 were annealed and ligated into BbsI-digested psgRNA(MS2).	This work
pNF366	P_{hU6}-sgRNA$_{ACE2\text{-}2}$<br><br>P_{hU6}-driven sgRNA complementary to a sequence in the exon 13 of human *ACE2* gene.<br><br>Oligonucleotides oNF652 and oNF653 were annealed and ligated into BbsI-digested psgRNA(MS2).	This work
pNF367	P_{hU6}-sgRNA$_{ACE2\text{-}3}$<br><br>P_{hU6}-driven sgRNA complementary to a sequence in the exon 14 of human *ACE2* gene.<br><br>Oligonucleotides oNF654 and oNF655 were annealed and ligated into BbsI-digested psgRNA(MS2).	This work
pNF368	P_{hU6}-sgRNA$_{PCSK9}$<br><br>P_{hU6}-driven sgRNA complementary to a sequence in the exon 1 of murine *PCSK9* gene.<br><br>Oligonucleotides oNF656 and oNF657 were annealed and ligated into BbsI-digested psgRNA(MS2).	This work
pNF372	P_{mPGK1}-RelA-pA<br><br>Mammalian expression plasmid encoding P_{mPGK1}-driven full length RelA.<br><br>RelA was first amplified from pRSV-p65 using oNF660 and oNF662 and second with oNF661 and oNF663 to remove internally occurring EcoRI site, following by PCR assembly using oNF660 and oNF663. The PCR fragment was digested with EcoRI/XbaI and ligated into pNF167 (EcoRI/XbaI)	This work
pNF377	P_{mPGK1}-HCVp-fLuc$_{CS\text{-}K491}$-pA<br><br>Mammalian expression plasmid encoding P_{mPGK1}-driven HCVp linked to N-terminal of fLuc containing HCVp CS.	This work

	HCVp was amplified from pNF251 using oNF468 and oNF358 and digested with EcoRI/BamHI. HCVp CS was introduced into fLuc by amplifying BB3-fLuc first with oNF374/oNF613 and second with oNF616/oNF375, followed by PCR assembly reaction using oNF374 and oNF375. The PCR fragment weas digested with BamHI/XbaI. Both fragments were ligated into pNF167 (EcoRI/XbaI).	
pNF378	P_{mPGK1}-HIVp-$fLuc_{CS\text{-}K491}$-pA<br><br>Mammalian expression plasmid encoding P_{mPGK1}-driven HIVp linked to N-terminal of fLuc containing HIVp CS.<br><br>HIVp was amplified from pNF212 using oNF376 and oNF358 and digested with EcoRI/BamHI. HIVp CS was introduced into fLuc by amplifying BB3-fLuc first with oNF374/oNF659 and second with oNF658/oNF375 followed by PCR assembly reaction using oNF374 and oNF375. PCR fragment was digested with BamHI/XbaI. Both fragments were ligated into pNF167 (EcoRI/XbaI).	This work
pNF379	P_{mPGK1}-$RelA_{N\text{-}term}$-$HRVp_{\Delta Q182}$-CS-$RelA_{C\text{-}term}$-pA<br><br>Mammalian expression plasmid encoding P_{mPGK1}-driven RelA modified to contain $HRVp_{\Delta Q182}$ and CS.<br><br>$RelA_{N\text{-}term}$ was amplified from pNF372 using oNF660 and oNF664 and $HRVp_{\Delta Q182}$-CS was amplified from pNF288 using oNF408 and oNF357, followed by PCR assembly reaction with oNF660 and oNF357. The PCR fragment was digested with EcoRI/BamHI. $RelA_{C\text{-}term}$ was amplified from pNF372 using oNF665 and oNF663 and digested with BamHI/XbaI. Both fragments were ligated into pNF167 (EcoRI/XbaI).	This work
pNF381	P_{mPGK1}-MyD88-pA<br><br>Mammalian expression plasmid encoding P_{mPGK1}-driven MyD88.<br><br>MyD88 was amplified from MYD88 using oNF669 and oNF670. The PCR product was digested with EcoRI/XbaI and ligated into pNF167 (EcoRI/XbaI).	This work
pNF384	P_{mPGK1}-$RelA_{N\text{-}term}$-HRVp-$RelA_{C\text{-}term}$-pA<br><br>Mammalian expression plasmid encoding P_{mPGK1}-driven RelA modified to contain HRVp.<br><br>$RelA_{N\text{-}term}$ was amplified from pNF372 using oNF660 and oNF664 and HRVp was amplified from pNF311 using oNF408 and oNF358, followed by PCR assembly reaction with oNF660 and oNF358. The PCR fragment was digested with EcoRI/BamHI. $RelA_{C\text{-}term}$ was amplified from pNF372 using oNF665 and oNF663 and digested with BamHI/XbaI. Both fragments were ligated into pNF167 (EcoRI/XbaI).	This work
pNF385	P_{mPGK1}-$HRVp_{\Delta Q182}$-$MyD88_{CS\text{-}G83}$-pA<br><br>Mammalian expression plasmid encoding P_{mPGK1}-driven MyD88 containing HRVp CS after G83 with N-terminally fused $HRVp_{\Delta Q182}$.<br><br>$HRVp_{\Delta Q182}$ was excised from pNF234 with EcoRI/BamHI. To introduce CS, MyD88 was first amplified from MYD88 using oNF689 and oNF671 and second with oNF672 and oNF670, followed by PCR assembly reaction using oNF689 and oNF670,	This work

	and digestion with BamHI/XbaI. Both fragments were ligated into pNF167 (EcoRI/XbaI).	
pNF386	P_{mPGK1}-HRV$p_{\Delta Q182}$-MyD88$_{CS\text{-}R188}$-pA<br><br>Mammalian expression plasmid encoding P_{mPGK1}-driven MyD88 containing HRVp CS after R188 with N-terminally fused HRV$p_{\Delta Q182}$.<br><br>HRV$p_{\Delta Q182}$ was excised from pNF234 with EcoRI/BamHI. To introduce CS, MyD88 was first amplified from MYD88 using oNF689 and oNF673 and second with oNF674 and oNF670, followed by PCR assembly reaction using oNF689 and oNF670 and digestion with BamHI/XbaI. Both fragments were ligated into pNF167 (EcoRI/XbaI).	This work
pNF387	P_{mPGK1}-HRV$p_{\Delta Q182}$-MyD88$_{CS\text{-}G201}$-pA<br><br>Mammalian expression plasmid encoding P_{mPGK1}-driven MyD88 containing HRVp CS after G201 with N-terminally fused HRV$p_{\Delta Q182}$.<br><br>HRV$p_{\Delta Q182}$ was excised from pNF234 with EcoRI/BamHI. To introduce CS, MyD88 was first amplified from MYD88 using oNF689 and oNF675 and second with oNF676 and oNF670, followed by PCR assembly reaction using oNF689 and oNF670 and digestion with BamHI/XbaI. Both fragments were ligated into pNF167 (EcoRI/XbaI).	This work
pNF388	P_{mPGK1}-MyD88$_{CS\text{-}G83}$-pA<br><br>Mammalian expression plasmid encoding P_{mPGK1}-driven MyD88 containing CS after G83.<br><br>To introduce CS, MyD88 was first amplified from MYD88 using oNF689 and oNF671 and second with oNF672 and oNF670, followed by PCR assembly reaction using oNF689 and oNF670, and digestion with BamHI/XbaI. The fragment was ligated into pNF220 (BamHI/XbaI).	This work
pNF389	P_{mPGK1}-MyD88$_{CS\text{-}R188}$-pA<br><br>Mammalian expression plasmid encoding P_{mPGK1}-driven MyD88 containing CS after R188.<br><br>To introduce CS, MyD88 was first amplified from MYD88 using oNF689 and oNF673 and second with oNF674 and oNF670, followed by PCR assembly reaction using oNF689 and oNF670 and digestion with BamHI/XbaI. The fragment was ligated into pNF220 (BamHI/XbaI).	This work
pNF390	P_{mPGK1}-MyD88$_{CS\text{-}G201}$-pA<br><br>Mammalian expression plasmid encoding P_{mPGK1}-driven MyD88 containing CS after G201.<br><br>To introduce CS, MyD88 was first amplified from MYD88 using oNF689 and oNF675 and second with oNF676 and oNF670, followed by PCR assembly reaction using oNF689 and oNF670 and digestion with BamHI/XbaI. The fragment was ligated into pNF220 (BamHI/XbaI).	This work
pNF411	P_{hCMV}_P_{T7}-RelA$_{N\text{-}term}$-HRVp-RelA$_{C\text{-}term}$-pA<br><br>Mammalian expression plasmid encoding P_{hCMV}_P_{T7}-driven RelA modified to contain HRVp.	This work

	P_{hCMV_PT7} was digested from pcDNA3.1(+) using MluI/EcoRI and ligated into pNF384 (MluI/EcoRI).	
pNF414	P_{mPGK1}-TetR-CS-VP64-pA<br><br>Mammalian expression plasmid encoding P_{mPGK1}-driven DNA binding domain TetR fused to transcription activation domain VP64 with fusion linker containing HRVp CS and NLS.<br><br>TetR-VP64 containing HRVp CS was amplified from pNF244 using oNF249 and oNF328 followed by digestion with EcoRI/XbaI. The fragment was ligated into pNF167 (EcoRI/XbaI).	This work
pNF415	$P_{NF\text{-}\kappa B}$-Citrine-pA<br><br>$P_{NF\text{-}\kappa B}$-driven citrine reporter protein expression vector.<br><br>$P_{NF\text{-}\kappa B}$ was excised from pYL43 with MluI/HindIII and ligated into pFS220 (MluI/HindIII)	This work
pNF416	P_{mPGK1}-TetR-VP64-pA<br><br>Mammalian expression plasmid encoding P_{mPGK1}-driven DNA binding domain TetR fused to transcription activation domain VP64 with fusion linker containing NLS.<br><br>TetR was amplified from pNF167 using oNF249 and oNF261, followed by digestion with EcoRI/BamHI. The fragment was ligated into pNF167 (EcoRI/BamHI).	This work
pNF417	ITR-O_{TetR}-$P_{hCMVmin}$-SEAP-sTRSV-pA:P_{mPGK1}-HRVp$_{\Delta Q182}$-TetR-CS-VP64-pA:P_{RPBSA}-PuroR-P2A-YPet-pA ITR<br><br>SB100X-specific transposon containing a O_{TetR}-$P_{hCMVmin}$ -driven SEAP expression unit, P_{mPGK1}-driven HRVp$_{\Delta Q182}$-TetR-CS-VP64 and a constitutive (puromycin resistance) PuroR and YPet expression unit.<br><br>O_{TetR}-$P_{hCMVmin}$-SEAP-sTRSV was excised from pNF293 with MluI/HindIII. P_{mPGK1}-HRVp$_{\Delta Q182}$-TetR-CS-VP64 was excised from pNF244 with MluI/HindIII. P_{RPBSA}-YPet-p2A-PuroR was excised from pTS1024 with MluI/HindIII and cloned into respective acceptor sites A1, A2 and A3 of pTS1107, respectively.	This work
pNF418	P_{mPGK1}-fLuc-pA<br><br>Mammalian expression plasmid encoding P_{mPGK1}-driven fLuc.<br><br>fLuc was excised from bb3-fLuc with EcoRI/XbaI and ligated into pNF167 (EcoRI/XbaI).	This work

Abbreviations

fLuc, firefly luciferase derived from *Photinus pyralis*
15gs, flexible linker containing 15x glycine and serine
sTRSV, engineered hammerhead ribozyme derived from the natural ribozyme from the satellite RNA of the tobacco ringspot virus
TetR, Tet repressor protein derived from *Escherichia coli* transposon Tn10
SEAP, human, placental secreted alkaline phosphatase
ss-nLuc, secreted version of nanoluc luciferase derived from deep sea shrimp *Oplophorus gracilirostris*
P_{TREBI}, bidirectional 3G tetracycline-responsive promoter
O_{TetR}, TetR operator consisting of 7 repeats of TetR binding sites
P_{hCMV}, human cytomegalovirus immediate early promoter
$P_{hCMVmin}$, minimal human cytomegalovirus immediate early promoter

P_{mPGK}, mouse phosphoglycerate promoter
VP64, 4 core repeats of VP16 transactivation domain
NLS, nuclear localization sequence
MCS, multiple cloning site
pA, polyadenylation signal
Env140ac, active ribozyme from environmental samples
3'UTR, three prime untranslated region

Supplementary Table S4. Oligonucleotides used for cloning in this study.

**Oligonucleotide name**	**Sequence**
oNF237	CGTGAGAATTCACCATGGCTTCAAACTTTACTCAGTTCGTG
oNF249	TGCTGGAATTCGCCACCATGTCCAGATTAGATAAAAGTAAAGTGATTAAC
oNF260	GGCTCCGGGGGCGGAGGCTCCGGAGGCGGTTCCAGATTAGATAAAAGTAAAGTGATTAACAGC
oNF261	GGGCCGGATCCACTTCCGGACCCACTTTCACATTTAAGTTGTTTTTC
oNF328	GGCCCTCTAGATGCATGTTACCTAGAGTTAATCAGCATGTCCAG
oNF352	GGGCCGGATCCTGGACCTTGAAACAGCACTTCGAGACTTCCGGACCCACTTTCAC
oNF353	GACCGAATTCGCCACCATGGGCCCCAATACCGAGTTC
oNF354	ACCGCCTCCGGAGCCTCCGCCCCCGGAGCCTCCGCCTCCGGAACCCTGCTTTTCGACGAAATATTGTTTC
oNF355	CTTAAATGTGAAAGTGGGTCCGGAAGTGGCCCCAATACCGAGTTC
oNF357	GGGCCGGATCCTGGACCTTGAAACAGCACTTCGAGACCGCCTCCGGAGCCTCC
oNF358	TGGGCCGGATCCACCGCCTCCGGAGCCTCC
oNF374	GAAGTGGATCCGGCGAAGACGCCAAAAACATAAAGAAAGG
oNF375	GGCCCTCTAGATGCATGTTACACGGCGATCTTTCCGC
oNF376	AGACCGAATTCGCCACCATGCCTCAGATCACTCTTTGGCAG
oNF377	ACCGCCTCCGGAGCCTCCGCCCCCGGAGCCTCCGCCTCCGGAACCAAAATTTAAAGTGCAGCCAATCTGAG
oNF378	GGGCCGGATCCTTGCAAGTTCTGAACAATGGGATAGTTTTGACTGACCTGTGAACTTCCGGACCCACTTTCAC
oNF379	GGACCCACTTTCACATTTAAGTTG
oNF380	CAACTTAAATGTGAAAGTGGGTCCGGAAGTCCTCAGATCACTCTTTGGCAG
oNF383	AGACCGAATTCGCCACCATGGGATCCGGCTTCCAGGCGGCCGAGCG
oNF384	TGGACCTTGAAACAGCACTTCGAGTCCGGAACCTCCCAGAAGTGCCTCAGCAATTTC
oNF385	CTCGAAGTGCTGTTTCAAGGTCCAGGCTCCGGGGCTGGCTGTGATCCTGAGC
oNF386	TGGACCTTGAAACAGCACTTCGAGTCCGGAACCCGGGGTGGTGCAGGACTG
oNF387	CTCGAAGTGCTGTTTCAAGGTCCAGGCTCCGGGCACCTCCACTCCATCCTGAAG
oNF388	TGGACCTTGAAACAGCACTTCGAGTCCGGAACCACCCAAGGACACCAAAAGC
oNF389	CTCGAAGTGCTGTTTCAAGGTCCAGGCTCCGGGGCTGATGTCAATGCTCAGGAG
oNF390	GGCCCTCTAGATGCATGTTATAACGTCAGACGCTGGCCTC
oNF403	ACCGCCTCCGGAGCCTCCGCCCCCGGAGCCTCCGCCTCCGGAACCCTTTTCGACGAAATATTGTTTCTTGAG
oNF408	GGTGGAGGCGGATCTGGAGGTGGAGGCTCAGGCCCCAATACCGAGTTCG
oNF409	GGTCTCGAAGTGCTGTTTCAAGGTCCAGGTGGCTCCGGGACGATGACGGAAAAAGAGATCG
oNF410	GCCACCTGGACCTTGAAACAGCACTTCGAGACCTCCGGAACCCTTTCCGTGCTCCAAAACAACAAC
oNF414	GGAGGTGGCGGAAGTGGCCCCAATACCGAGTTCG
oNF415	GTATTGGGGCCACTTCCGCCACCTCCGTAGATACCTGAGTTAGCGGC
oNF464	GCGGTGGATCCGGCGACAAGAAGTATTCTATCGGACTGG
oNF465	GCCTGGACCTTGAAACAGCACTTCGAGTCCGGAACCCACTTGGGCCTTCTGGATGTC
oNF466	GGACTCGAAGTGCTGTTTCAAGGTCCAGGCTCCGGGTCTGGACAAGGTGACTCACTGCAC

oNF467	GGCCCTCTAGATGCATGTTATACCTTTCTCTTCTTTTTTGGATCTACCTTTCTCTTCTT TTTTGGATCTACCTTTCTCTTCTTTTTTGGATCACTAGCAGCTCC
oNF468	GACCGAATTCGCCACCATGTCCGGGACAGGCTGCGTGGTCATAGTGGGCAGGATCG TCTTGTCCGGCTCCGGCACTTCCGCGCCCATC
oNF469	ACCGCCTCCGGAGCCTCCGCCCCCGGAGCCTCCGCCTCCGGAACCGAACTCCTGGT AGAGAACCTCCC
oNF471	GGGCCGGATCCGTGCTGAGAGCACTCTTCCATCTCATCGAACTCACTTCCGGACCC ACTTTCAC
oNF477	GAAAAACAACTTAAATGTGAAAGTGGGTCCGGAAGTGCGTCTGCAGGCATGGCC
oNF478	GGGCCGGATCCGTGCTGAGAGCACTCTTCCATC
oNF484	GGCCCTCTAGATGCATGTTACTTTTCGACGAAATATTGTTTCTTGAGTTGAGCGG
oNF495	GCGGTGGATCCGGCGACAAGAAGTATTCTATCGGACTGGACATCGGGACTAATAG
oNF496	CTCAGACTACGACGTGGACCACATCGTCCCTCAGAGC
oNF497	GCTCTGAGGGACGATGTGGTCCACGTCGTAGTCTGAG
oNF498	CGAGTTTTCTAAACGGGTCATTCTCGCTGATG
oNF499	CATCAGCGAGAATGACCCGTTTAGAAAACTCG
oNF503	AGACCGAATTCGCCACCATGAGCGGCTCCTCTGCCAGCTTC
oNF504	CACGGACTCGCCTTGTGGCATCCCCCCCAAG
oNF505	CTTGGGGGGGATGCCACAAGGCGAGTCCGTG
oNF506	GCTGATGAGCGGATTCTTAAGCCGCACGCC
oNF507	GGCGTGCGGCTTAAGAATCCGCTCATCAGC
oNF508	TGAGCCTCCACCTCCAGATCCGCCTCCACCGCCTCCATGGGTGGGGCTG
oNF509	GACCGAATTCGCCACCATGCCGCCGGCGGATCACCCCTC
oNF510	TGAGCCTCCACCTCCAGATCCGCCTCCACCCTGAGCTGAGCGCTGGGAG
oNF530	GGACTCGAAGTGCTGTTTCAAGGTCCAGGCTCCGGGATCACCAAGGCCCCTCTTTC AGC
oNF531	GCCTGGACCTTGAAACAGCACTTCGAGTCCGGAACCTTCAGTGTTCACGCGGAGAA TGTC
oNF532	GGACTCGAAGTGCTGTTTCAAGGTCCAGGCTCCGGGAAGCCCGCATTCCTGTCAGG C
oNF533	GCCTGGACCTTGAAACAGCACTTCGAGTCCGGAACCCCTCATCCCTTCGGTGACAT ATTTC
oNF534	TGAGCCTCCACCTCCAGATCCGCCTCCACCCTTTCCGTGCTCCAAAACAACAAC
oNF539	TCCAGGATCCGGCCCAAAGAAGAAACGGAAGGTGGGAAGTGGTCTGGGGAGCCAG CCTTACTAC
oNF540	GCCCTCTAGATTACGTCTGATTTCTGGCAGGAGG
oNF545	CGGAGGCTCCGGGGGCGGAGGCTCCGGAGGCGGTACGATGACGGAAAAAGAGATC GTG
oNF601	AGACCGAATTCGCCACCATGAACGCCCCCGAGCGGCAG
oNF613	ACCTCCGGAACCCTTTCCGTG
oNF616	TTGTTTTGGAGCACGGAAAGGGTTCCGGAGGTGAGGATGTCGTGCCATGTTCTATG GGCTCTGGTGGCTCCGGGACGATG
oNF658	TCACAGGTCAGTCAAAACTATCCCATTGTTCAGAACTTGCAAGGTGGCTCCGGGAC GATGACGGAAAAAGAGATCGTG
oNF659	TTGCAAGTTCTGAACAATGGGATAGTTTTGACTGACCTGTGAACCTCCGGAACCCTT TCCGTGCTCCAAAACAACAACG
oNF660	AGACCGAATTCGCCACCATGGACGAACTGTTCCCCCTCATC
oNF661	CAGTGAGCCCATGGAGTTCCAGTACCTGCC
oNF662	GGCAGGTACTGGAACTCCATGGGCTCACTG
oNF663	GGCCCTCTAGATTAGGAGCTGATCTGACTCAGCAG
oNF664	TGAGCCTCCACCTCCAGATCCGCCTCCACCGCTGAAAGGACTCTTCTTCATGATGC
oNF665	GTCCAGGATCCGGCCCAAAGAAGAAACGGAAGGTGGGAAGTGGTGGACCCACCGA CCCCCGGCC
oNF669	AGACCGAATTCGCCACCATGGCTGCAGGAGGTCCCGG
oNF670	GCCCTCTAGATGCATGTTAGGGCAGGGACAAGGCCTTGG
oNF671	TGGACCTTGAAACAGCACTTCGAGTCCGGAACCGCCAGGGCGTCCCTGCCAG
oNF672	CTCGAAGTGCTGTTTCAAGGTCCAGGCTCCGGGGCCTCTGTAGGCCGACTGC
oNF673	GCCTGGACCTTGAAACAGCACTTCGAGTCCGGAACCTCGATAGTTTGTCTGTTCCA GTTGC
oNF674	GGACTCGAAGTGCTGTTTCAAGGTCCAGGCTCCGGGCTGAAGTTGTGTGTGTCTGA CCG

oNF675	GCCTGGACCTTGAAACAGCACTTCGAGTCCGGAACCGCCAGGCAGGACATCGCGG
oNF676	GACTCGAAGTGCTGTTTCAAGGTCCAGGCTCCGGGACCTGTGTCTGGTCTATTGCTA G
oNF689	GCGGTGGATCCGGCGCTGCAGGAGGTCCCGGC

Supplementary Table S5. Oligonucleotides used for sgRNA construction.

Oligonucleotide name	Sequence
oNF593-sgRNA(EMX1)_for	GCCATCCCCTTCTGTGAATGTTAG
oNF594-sgRNA(EMX1)_rev	GGAGATTGGAGACACGGAGAGCAG
oNF638-sgRNA(VEGFA)-1_for	CACCGCCTGGAGTGTGTGCCCACTG
oNF639-sgRNA(VEGFA)-1_rev	AAACCAGTGGGCACACACTCCAGGC
oNF640-sgRNA(VEGFA)-2_for	CACCGATGCGGATCAAACCTCACCA
oNF641-sgRNA(VEGFA)-2_rev	AAACTGGTGAGGTTTGATCCGCATC
oNF642-sgRNA(VEGFA)-3_for	CACCGCCTGTGGGCCTTGCTCAGAG
oNF643-sgRNA(VEGFA)-3_rev	AAACCTCTGAGCAAGGCCCACAGGC
oNF644-sgRNA(TNFRSF1A)-1_for	CACCGAAAGTTGGGACAGTCACCGG
oNF645-sgRNA(TNFRSF1A)-1_rev	AAACCCGGTGACTGTCCCAACTTTC
oNF646-sgRNA(TNFRSF1A)-2_for	CACCGGGTGGCACCACCCTATCAGG
oNF647-sgRNA(TNFRSF1A)-2_rev	AAACCCTGATAGGGTGGTGCCACCC
oNF648-sgRNA(TNFRSF1A)-3_for	CACCGCTTTGCGGCTCCCCGCAGAG
oNF649-sgRNA(TNFRSF1A)-3_rev	AAACCTCTGCGGGGAGCCGCAAAGC
oNF650-sgRNA(ACE2)-1_for	CACCGATCAGCCATATGGAAACAGG
oNF651-sgRNA(ACE2)-1_rev	AAACCCTGTTTCCATATGGCTGATC
oNF652-sgRNA(ACE2)-2_for	CACCGTGTCAAGCAGCTAAACATGA
oNF653-sgRNA(ACE2)-2_rev	AAACTCATGTTTAGCTGCTTGACAC
oNF654-sgRNA(ACE2)-3_for	CACCGGGCTTGGAAAATCAGAACCC
oNF655-sgRNA(ACE2)-3_rev	AAACGGGTTCTGATTTTCCAAGCCC
oNF656-sgRNA(PCSK9)_for	CACCGGGCTGATGAGGCCGCACATG
oNF657-sgRNA(PCSK9)_rev	AAACCATGTGCGGCCTCATCAGCCC

Supplementary Table S6. Synthetic DNA fragments used in this study

**Fragment name**	**Sequence**
DNA_twist_ HRVp	ATGGGCCCCAATACCGAGTTCGCGCTCTCTCTTCTGAGGAAAAATATTATGACAA TCACGACTTCCAAGGGGGAGTTTACCGGATTGGGTATACATGACCGAGTTTGTGT AATCCCTACGCATGCTCAACCTGGAGATGACGTTTTGGTTAACGGGCAAAAGATC CGAGTTAAGGATAAGTATAAGCTCGTTGATCCTGAAAACATCAACCTCGAACTGA CAGTCCTGACACTGGACCGCAATGAAAAATTTCGCGACATTAGAGGTTTTATCTC TGAAGACCTTGAAGGTGTCGATGCCACGTTGGTCGTTCACAGCAATAACTTTACG AATACAATCCTTGAGGTCGGACCTGTAACTATGGCGGGGCTCATAAATCTGAGTT CTACGCCTACCAACAGAATGATTAGATACGATTATGCTACCAAGACCGGCCAATG CGGTGGTGTCCTGTGTGCTACGGGCAAGATATTCGGGATTCATGTAGGTGGCAAC GGGAGACAGGGCTTTTCCGCTCAACTCAAGAAACAATATTTCGTCGAAAAGCAG

Supplementary Table S7. Oligonucleotides used to generate amplicons for next-generation Sequencing.

EMX1_NGS_for	ACACTCTTTCCCTACACGACGCTCTTCCGATCTGGAGGACAAAGTACA AACGGC
EMX1_NGS_rev	GTGACTGGAGTTCAGACGTGTGCTCTTCCGATCATCGATGTCCTCCCC ATTGG
VEGFA-1_NGS_for	ACACTCTTTCCCTACACGACGCTCTTCCGATCTGTGTGCCCCTGATGC GATG
VEGFA-1_NGS_rev	GTGACTGGAGTTCAGACGTGTGCTCTTCCGATCGGGACCACCTGTTCC CAAAGT

VEGFA-2_ NGS _for	ACACTCTTTCCCTACACGACGCTCTTCCGATCTTGGCTGGGTCACTAACCTCT
VEGFA-2_ NGS _rev	GTGACTGGAGTTCAGACGTGTGCTCTTCCGATCCCTGCAGCCACTTGCTGATA
VEGFA-3_ NGS _for	ACACTCTTTCCCTACACGACGCTCTTCCGATCTAGCTGCGGACATGTTAGGGG
VEGFA-3_ NGS _rev	GTGACTGGAGTTCAGACGTGTGCTCTTCCGATCCAGGAACATTTACACGTCTGCG
TNFRSF1A-1_NGS_for	ACACTCTTTCCCTACACGACGCTCTTCCGATCTTGGGCTTCAGTCCCGTGC
TNFRSF1A-1_NGS_rev	GTGACTGGAGTTCAGACGTGTGCTCTTCCGATCTGTCCTCCCACTTCTGAAGG
TNFRSF1A-2_NGS_for	ACACTCTTTCCCTACACGACGCTCTTCCGATCTTGCCCAGTTCCACCTTCAC
TNFRSF1A-2_NGS_rev	GTGACTGGAGTTCAGACGTGTGCTCTTCCGATCAGAAACTCACTGTCTAGGCTCTG
ACE2-1_NGS_for	ACACTCTTTCCCTACACGACGCTCTTCCGATCTTTCCGTCTGAATGACAACAGC
ACE2-1_NGS_rev	GTGACTGGAGTTCAGACGTGTGCTCTTCCGATCTCCGATCTCTGATCCCAGTGAA
ACE2-2_NGS_for	ACACTCTTTCCCTACACGACGCTCTTCCGATCTTGCTTTGTCTCCTGTGCAGATA
ACE2-2_NGS_rev	GTGACTGGAGTTCAGACGTGTGCTCTTCCGATCTAGGAGAGGTTCAACATTTTGAGGT
ACE2-3_NGS_for	ACACTCTTTCCCTACACGACGCTCTTCCGATCTACATCTGGAACCCCTCAAAAGG
ACE2-3_NGS_rev	GTGACTGGAGTTCAGACGTGTGCTCTTCCGATCCTCAAAGTAGTTGAGCAGTGGC

Supplementary Figures

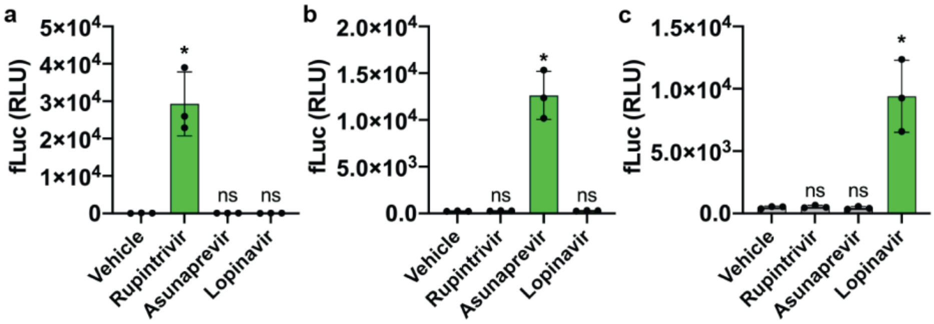

Figure S1: Responsiveness of iCROP-fLuc to different protease inhibitors. Bioluminescence intensity of HEK293T cells transfected with fLuc N-terminally fused to **a)** HRVp, **b)** HCVp or **c)** HIVp and bearing the corresponding cleavage sites downstream of residue K491. Following transfection, cells were incubated with each protease inhibitor (1 µM) for 24 h before measuring luminescence intensity. Data are shown as mean ± s.d., with individual data points (*n* = 3 biological replicates). Statistical significance was calculated by means of Welch's two-tailed *t*-test, $*P < 0.05$, $**P < 0.01$, $***P < 0.001$, $****P < 0.0001$, ns, not significant.

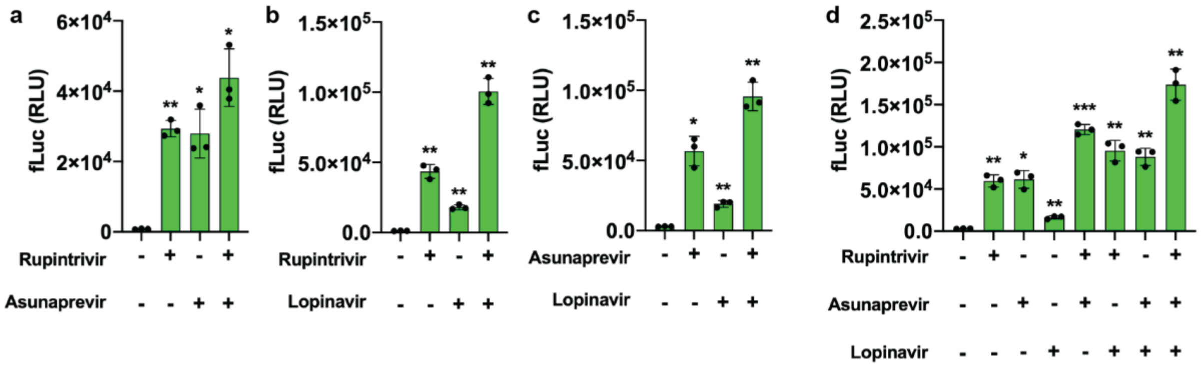

Figure S2: Two- and three-input OR logic gates. FLuc activity in cells co-expressing **a)** iCROP-fLuc$_{HRVp}$ and iCROP-fLuc$_{HCVp}$ (pNF247 and pNF377), **b)** iCROP-fLuc$_{HRVp}$ and iCROP-fLuc$_{HIVp}$ (pNF247 and pNF378), **c)** iCROP-fLuc$_{HCVp}$ and iCROP-fLuc$_{HIVp}$ (pNF377 and pNF378) and **d)** iCROP-fLuc$_{HRVp}$, iCROP-fLuc$_{HCVp}$ and iCROP-fLuc$_{HIVp}$ (pNF247, pNF377 and pNF378). Following transfection, cells were incubated with the indicated combination of protease inhibitors (1 µM) for 24 h before measuring the luminescence intensity. Data are shown as mean ± s.d., with individual data points (n = 3 biological replicates). Statistical significance was calculated by means of Welch's two-tailed t-test, *$P < 0.05$, **$P < 0.01$, ***$P < 0.001$, ****$P < 0.0001$, ns, not significant.

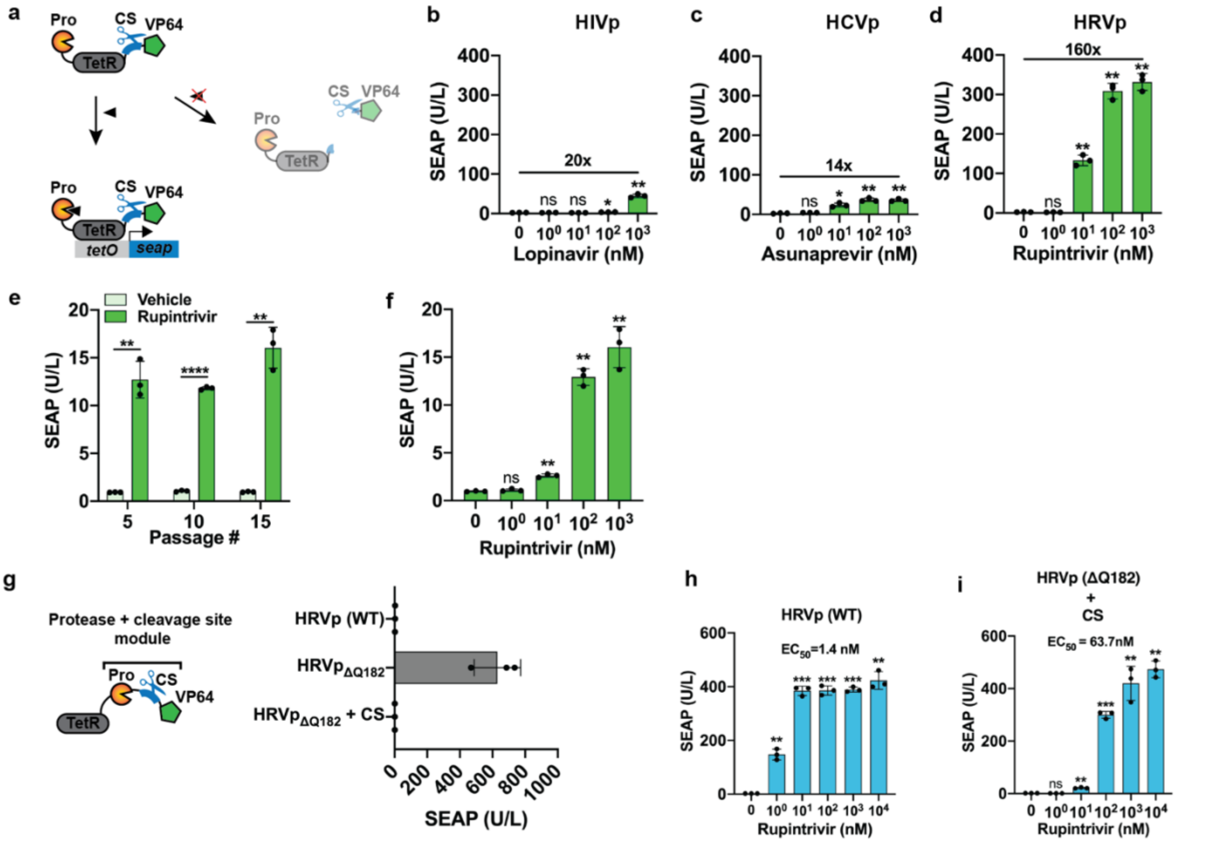

Figure S3: Performance of different iCROP systems applied to the multi-domain protein TetR-VP64. a) Scheme of TetR-VP64 modified to contain a protease cleavage site in the linker between TetR and VP64 and the corresponding protease (HIVp, HCVp or HRVp) fused to its N-terminus. **b-d)** SEAP secretion from HEK293T cells expressing each TetR-VP64 protease system, and treated for 24 h with different concentrations of the corresponding inhibitors. **e)** SEAP secretion by HEK293T cells with genomic integration of the expression cassettes O$_{TetR}$-P$_{hCMVmin}$-SEAP and P$_{mPGK}$-HRVp$_{ΔQ182}$-TetR-CS-VP64 (pNF417). The HEK-iCROP cells were

routinely passaged every two to three days and assessed for inducibility at the indicated passage numbers, by incubation with rupintrivir (1 µM) or vehicle for 24 h before SEAP measurement. **f)** Dose-dependent SEAP expression by HEK-iCROP cells after 15 passages. Cells were treated with the indicated rupintrivir concentrations before assessing SEAP expression levels. **g**) Effect of HRVp variant and CS module on inactivation of the synthetic transcription factor (left). The C-terminus of HRVp acts as a cleavage site and the deletion of the last amino acid (Q182) creates fully active TetR-VP64 that cannot be inactivated. Addition of the CS (Supplementary Table S2) to the $HRVp_{\Delta Q182}$ enables cleavage and TetR-VP64 inactivation (right). **h-i**) SEAP secretion from HEK293T cells co-transfected with TetR-responsive SEAP expression plasmid and constitutive expression of TetR-VP64 bearing either h) wt HRVp (EC_{50} = 1.4 nM) or i) $HRVp_{\Delta Q182}$ and CS module (EC_{50} = 63.7 nM) in the linker between TetR and VP64. The cells were treated for 24 h with the specified rupintrivir concentrations. Numbers above the lines in b-d indicate fold induction, calculated by dividing SEAP activity at the highest concentration by SEAP activity in untreated cells. Data in panels b-i are shown as mean ± s.d., with individual data points (*n* = 3 biological replicates). Statistical significance was calculated by means of Welch's two-tailed *t*-test, $*P < 0.05$, $**P < 0.01$, $***P < 0.001$, $****P < 0.0001$, ns, not significant.

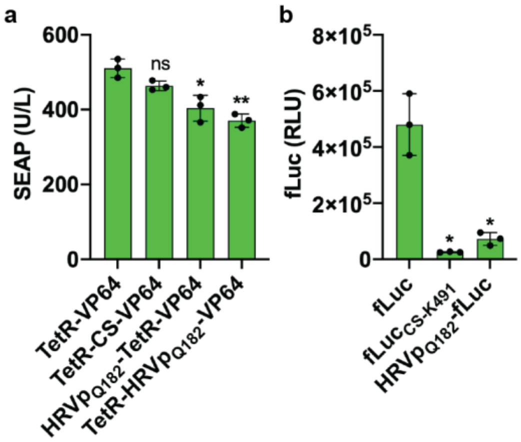

Figure S4: Effect of modifications on TetR-VP64 and fLuc activities. a) SEAP secretion by cells co-transfected with O_{TetR}-$P_{hCMVmin}$-SEAP (pNF293) and with constitutive expression of either unmodified TetR-VP64 or TetR-VP64 modified as indicated (either with a cleavage site or the $HRV_{\Delta Q182}$ protease between the TetR and VP64 domains or the $HRV_{\Delta Q182}$ protease N-terminally fused to TetR). SEAP expression levels were quantified 48 h post-transfection. **b)** Luminescence of cells transfected with unmodified fLuc or fLuc bearing the HRV cleavage site downstream of K491, either without or with the $HRV_{\Delta Q182}$ protease N-terminally fused. Following transfection, the luminescence intensity was quantified 48 h thereafter. Data are shown as mean ± s.d., with individual data points (*n* = 3 biological replicates). Statistical significance was calculated by means of Welch's two-tailed *t*-test, $*P < 0.05$, $**P < 0.01$, $***P < 0.001$, $****P < 0.0001$, ns, not significant.

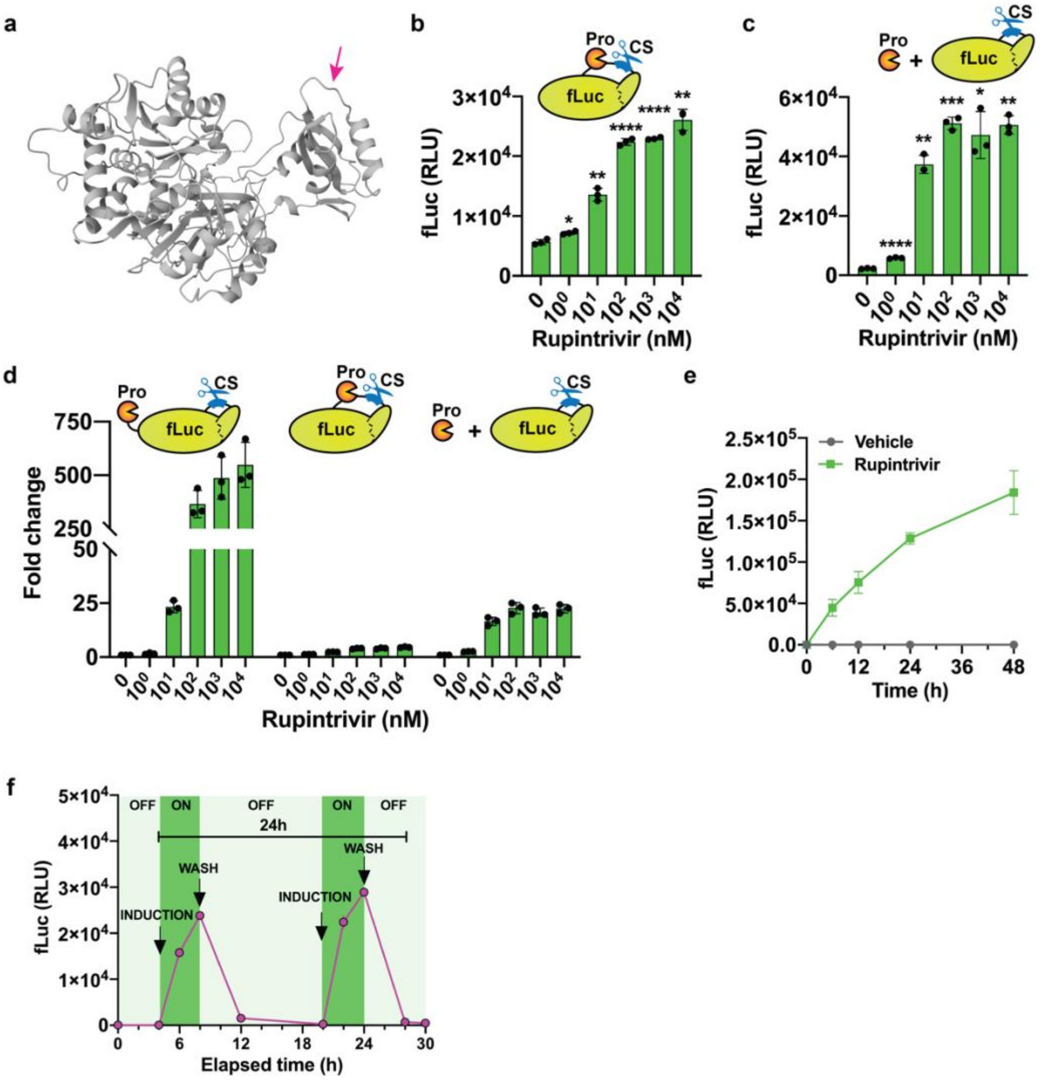

Figure S5: Assessment of various iCROP-fLuc topologies, kinetics and reversibility. a) fLuc 3D structure (PDB: 1BA3). The arrow points to residue K491, where the CS was placed. **b)** Bioluminescence intensity of HEK293T cells transfected with fLuc harboring both HRVp and CS downstream of K491, and incubated with the indicated rupintrivir concentrations for 24 h. **c)** Bioluminescence intensity of HEK293T cells co-transfected with fLuc bearing the CS downstream of K491 and a separate plasmid encoding HRVp. The cells were then incubated with the indicated rupintrivir concentrations for 24 h. **d)** Comparison of the three strategies for control of fLuc activity based on rupintrivir inhibition of HRVp. The fold change was calculated by dividing the fLuc activity at each rupintrivir concentration by the fLuc activity of untreated cells. **e)** Kinetics of iCROP-fLuc. Time-course analysis of fLuc luminescence in HEK293T cells transfected with iCROP-fLuc (P_{mPGK}-HRVp$_{\Delta Q182}$-fLuc$_{CS\text{-}K491}$-pA) and incubated for 48 h either in the absence or presence of rupintrivir (1 µM). **f)** iCROP-fLuc reversibility. HEK293T cells expressing iCROP-fLuc were alternated between rupintrivir-containing (100 nM) and rupintrivir-free media. Each rupintrivir induction lasted 4 h, followed by 12 h in rupintrivir-free medium. fLuc intensity was measured at the indicated time points. Data in panels b-f are shown as mean ± s.d., with individual data points in panels b-d (n = 3 biological replicates). Statistical significance was calculated by means of Welch's two-tailed t-test, *$P < 0.05$, **$P < 0.01$, ***$P < 0.001$, ****$P < 0.0001$, ns, not significant.

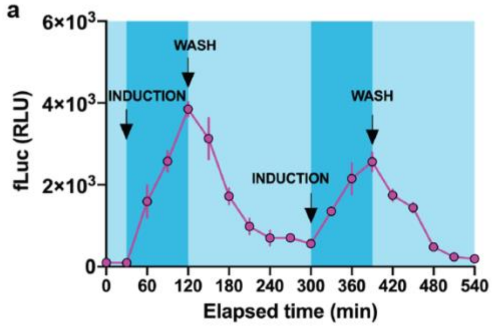

Figure S6: Reversibility of mRNA-delivered iCROP-fLuc. HEK293T cells expressing iCROP-fLuc delivered as mRNA were alternated between rupintrivir-containing (100 nM) and rupintrivir-free media, starting at 4 h post-transfection. Each rupintrivir induction lasted 90 min, followed by 180 min in rupintrivir-free medium. fLuc luminescence intensity was measured at the indicated time points. Data are shown as mean ± s.d. (*n* = 3 biological replicates).

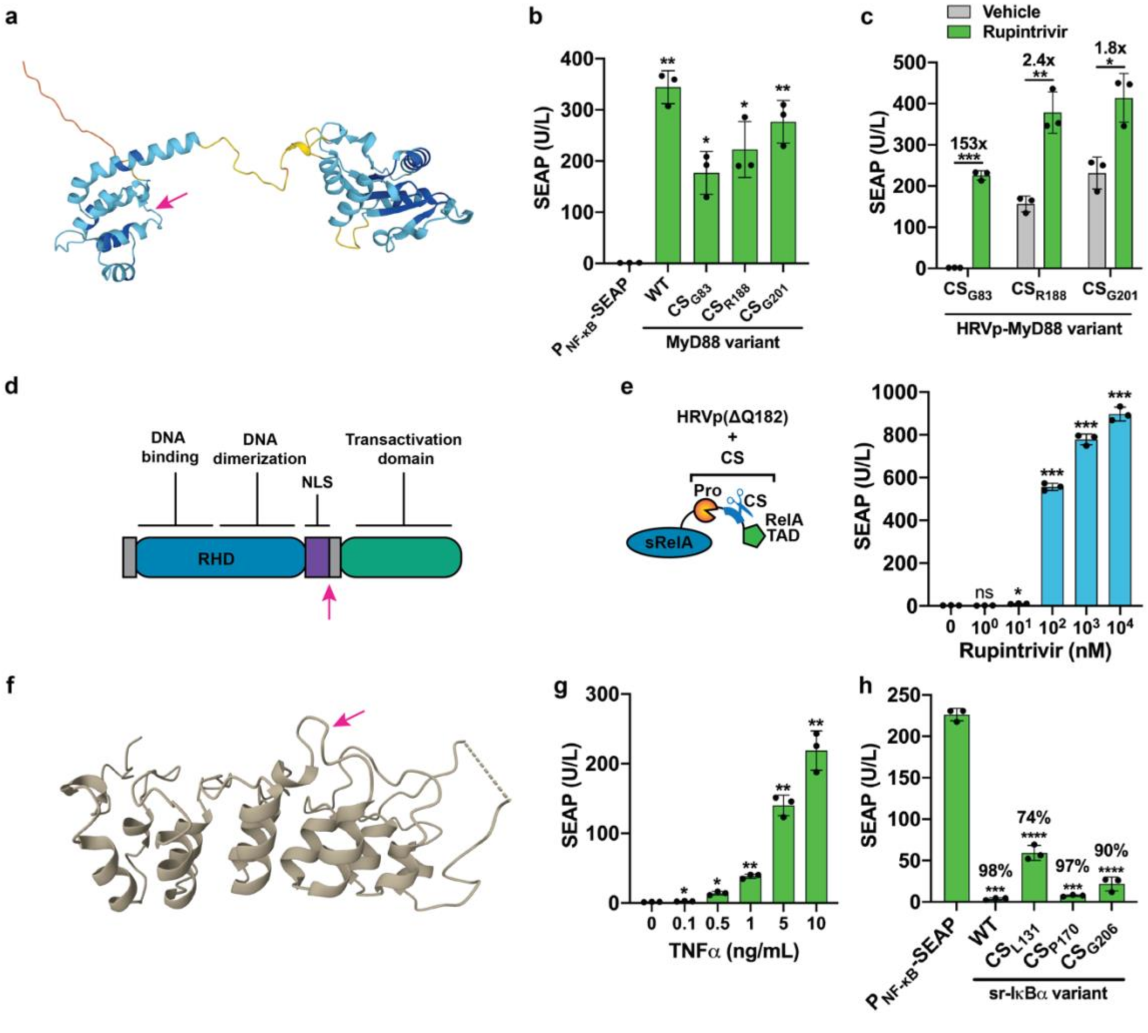

Figure S7: Design of inducible NF-κB regulators. **a)** Predicted 3D structure of MyD88 (AlphaFold: AF-Q99836-F1). The arrow points to residue G83, selected as the best location to place the CS. **b)** NF-κB activation by overexpression of wild-type (WT) and CS-modified MyD88 variants. SEAP levels were analyzed 36 h after co-transfection of HEK293T cells with constitutive expression of each MyD88 variant and SEAP under an NF-κB-responsive promoter ($P_{NF\text{-}\kappa B}$-SEAP-pA). Cells transfected with the reporter only were included as a negative control.

c) NF-κB signaling activation by MyD88 modified with CS in different positions and HRVp fused to the N' terminus in response to rupintrivir (1 μM). Numbers above bars indicate fold induction between rupintrivir-treated and untreated cells. **d)** Scheme of the RelA structure, in which the RHD domain responsible for DNA binding and dimerization is predicted to span between P19 and Y306 and the transactivation domain between P342 and A544. The arrow points to residue S319 downstream of the nuclear localization signal (NLS), where the wt HRVp with CS was placed. **e)** Regulation of RelA activity using the $HRVp_{\Delta Q182}$ and CS variants (left). NF-κB activation by inducible RelA in response to rupintrivir (right). **f)** 3D structure of IκBα (PDB: 1IKN). The arrow points to residue P170, selected as the best location to place the CS. **g)** NF-κB activation in response to TNFα treatment. Cells transfected with $P_{NF\text{-}\kappa B}$-SEAP-pA were challenged with the indicated TNFα concentrations for 24 h before SEAP analysis. **h)** NF-κB repression by overexpression of non-modified and CS-modified sr-IκBα variants. Cells were co-transfected with the sr-IκBα variants together with $P_{NF\text{-}\kappa B}$-SEAP-pA and treated with TNFα (10 ng/mL) to activate NF-κB signaling. SEAP expression levels were measured 24 h thereafter. Numbers above the bars indicate suppression level of sr-IκBα variants relative to non-suppressed NF-κB signaling. Data in panels b,c,e,g,h are shown as mean ± s.d., with individual data points (*n* = 3 biological replicates). Statistical significance was calculated by means of Welch's two-tailed *t*-test, *$P < 0.05$, **$P < 0.01$, ***$P < 0.001$, ****$P < 0.0001$, ns, not significant.

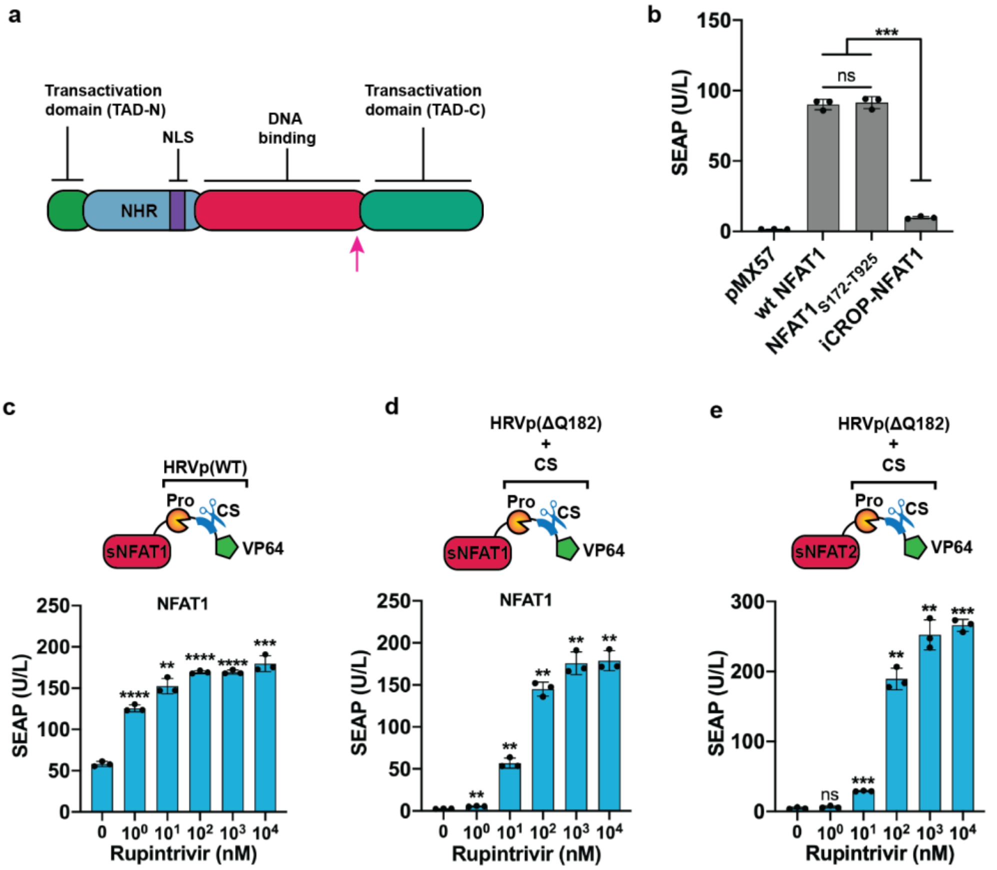

Figure S8: Design and optimization of inducible NFAT regulators. a) Schematic illustration of representative NFAT structure. The arrow points to the region of the DNA binding domain where the HRVp and CS were placed. **b)** Activation of SEAP expression from an NFAT promoter (pMX57, P_{3xNFAT}-SEAP-pA) in cells co-transfected with different NFAT1 variants, namely wt NFAT1, truncated $NFAT1_{S172\text{-}T925}$, and iCROP-NFAT1. Cells transfected with

pMX57 alone were included as a negative control. **c,d**) Activation of engineered NFAT1 bearing the **c)** wt HRVp (which has a cleavage site at its C-terminus) or **d)** HRVp$_{\Delta Q182}$ + CS module upstream of VP64. SEAP levels were determined after 24 h treatment with rupintrivir. **e)** Activation of NFAT2 modified with the HRVp$_{\Delta Q182}$ + CS module upstream of VP64 (which replaced the native transactivation domain. Data in panels b – e are shown as mean ± s.d., with individual data points (n = 3 biological replicates). Statistical significance was calculated by means of Welch's two-tailed t-test, *$P < 0.05$, **$P < 0.01$, ***$P < 0.001$, ****$P < 0.0001$, ns, not significant.

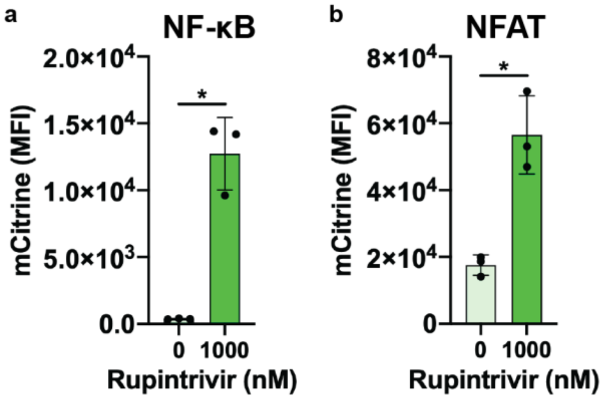

Figure S9: Regulation of NF-κB and NFAT pathways in Jurkat T cells using iCROP. a) Activation of NF-κB pathway in cells electroporated with pNF411 and pNF415. **b)** Activation of NFAT pathway in cells electroporated with pNF287 and pFS220. Cells were induced with rupintrivir (1 µM) for 24 h before measuring citrine fluorescence intensity. Data are shown as mean ± s.d., with individual data points (n = 3 biological replicates). Statistical significance was calculated by means of Welch's two-tailed t-test, *$P < 0.05$, **$P < 0.01$, ***$P < 0.001$, ****$P < 0.0001$, ns, not significant.

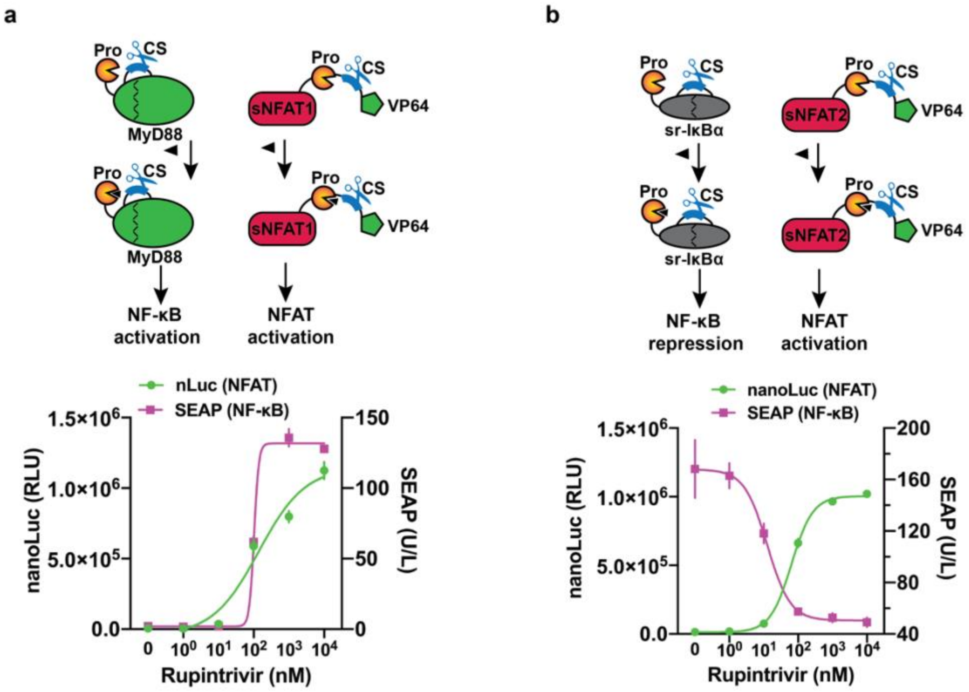

Figure S10: Multiplexing NF-κB and NFAT regulation with iCROP. a) Schematic illustration of iCROP-based NF-κB and NFAT co-activation in response to rupintrivir (top). HEK293T cells were co-transfected with iCROP-MyD88, P$_{NF\text{-}\kappa B}$-SEAP, iCROP-NFAT1 and

P_{3xNFAT}-nLuc-pA before titrating rupintrivir and determining nLuc and SEAP levels in the supernatant, 24 h thereafter (bottom). **b)** Schematic illustration of simultaneous iCROP-based NF-κB repression and NFAT activation in response to rupintrivir (top). Cells co-transfected with iCROP-sr-IκBα, $P_{NF-\kappa B}$-SEAP-pA, iCROP-NFAT2 and P_{3xNFAT}-nLuc-pA were challenged with TNFα (10ng/mL) to trigger NF-κB signaling and the indicated rupintrivir concentrations to activate both sr-IκBα and NFAT2 for 24 h before SEAP and nLuc analysis. Data are shown as mean ± s.d., (n = 3 biological replicates).

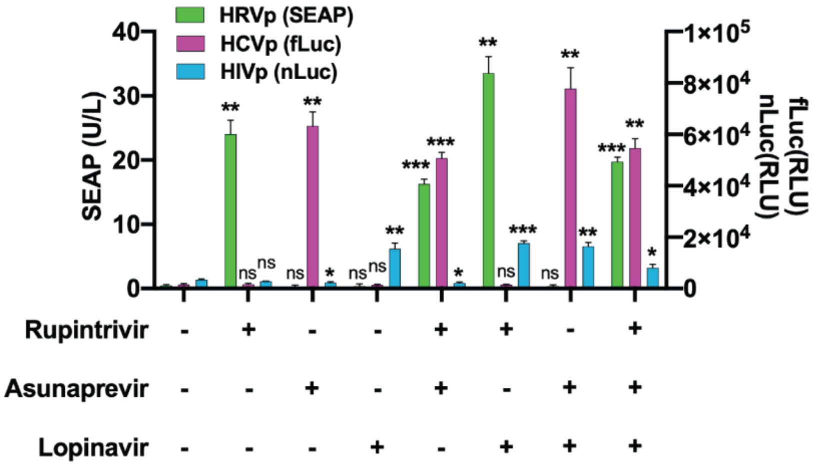

Figure S11: Simultaneous regulation of three proteins by iCROP. HEK293T cells were transfected with a combination of plasmids to report on the activity of three different proteins controlled by iCROP systems, namely SEAP expression controlled by iCROP-NFAT1$_{HRVp}$ (pNF287 and pMX57) or iCROP-fLuc$_{HCVp}$ (pNF377), and nLuc expression controlled by iCROP-TetR-VP64$_{HIVp}$ (pNF214 and pNF151). Following transfection, cells were incubated in the presence of all possible combinations of rupintrivir, asunaprevir and lopinavir (1 µM) for 24 h before measuring the activity of reporter proteins. Data are shown as mean ± s.d. (n = 3 biological replicates). Statistical significance was calculated by means of Welch's two-tailed t-test, *$P < 0.05$, **$P < 0.01$, ***$P < 0.001$, ****$P < 0.0001$, ns, not significant.

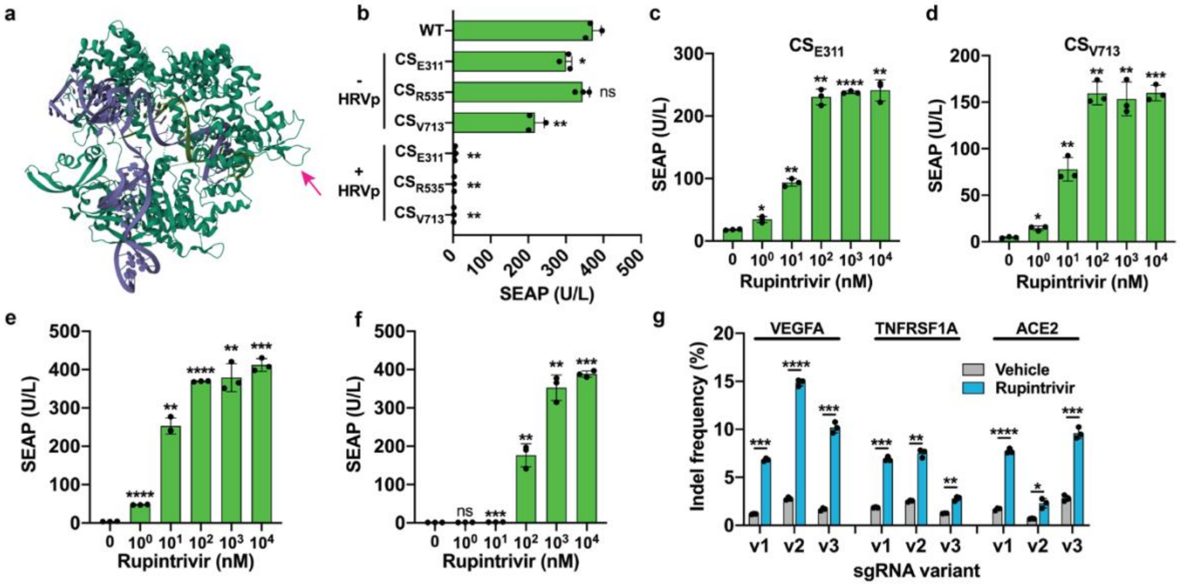

Figure S12: Design and validation of the CRISPR/(d)Cas9-based iCROP systems. **a)** 3D structure of Cas9 (PDB: 4OO8). The arrow points to the best screened location to place the CS (residue R535). **b)** Transcription activation performance of CS-modified dCas9 variants without and with N-terminally fused HRVp. HEK293T cells were transfected with wt dCas9 or CS-modified dCas9 variant with or without HRVp together with sgRNA(P_{INS})-MS2, MCP-p65$_{TA}$-HSF1$_{TA}$ and P_{INS}-SEAP 36 h before SEAP expression analysis. **c,d)** Rupintrivir-induced activation of iCROP-dCas9 modified with CS in position c) E311 or d) V713 in cells treated with rupintrivir for 24 h before determining SEAP levels. **e,f)** Rupintrivir-dependent transcription activation of the SEAP reporter gene in cells co-transfected with dCas9, sgRNA(P_{INS})-MS2, P_{INS}-SEAP and iCROP-MCP$_{VP64}$, modified with e) wt HRVp or f) HRVp$_{\Delta Q182}$ + CS module. **g)** iCROP-Cas9-induced indel frequencies of sgRNA variants targeting various endogenous genomic loci. Cells were transfected with iCROP-Cas9 together with an sgRNA variant and treated with rupintrivir (1 μM) or vehicle for 48 h before mutation rate analysis by NGS. Data are shown as mean ± s.d., with individual data points (n = 3 biological replicates). Statistical significance was calculated by means of Welch's two-tailed t-test, *$P < 0.05$, **$P < 0.01$, ***$P < 0.001$, ****$P < 0.0001$, ns, not significant.

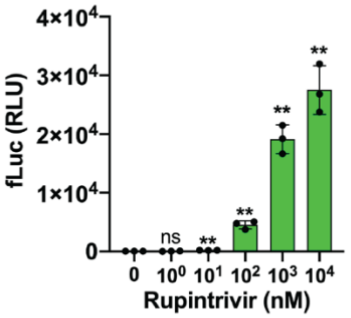

Figure S13: Performance of iCROP-fLuc in HepG2 liver cell line. Bioluminescence intensity of HepG2 cells transfected with iCROP-fLuc and treated with the indicated rupintrivir concentrations for 24 h. Data are shown as mean ± s.d., with individual data points (n = 3 biological replicates). Statistical significance was calculated by means of Welch's two-tailed t-test, *$P < 0.05$, **$P < 0.01$, ***$P < 0.001$, ****$P < 0.0001$, ns, not significant.

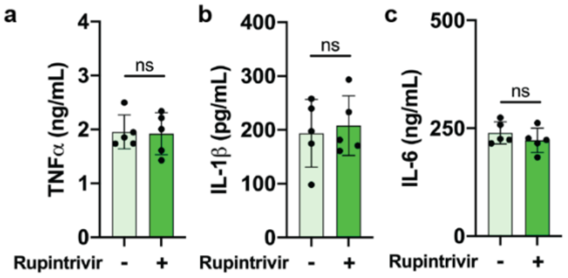

Figure S14: Effect of rupintrivir on TNFα, IL-1β and IL-6 *in vivo*. Blood levels of **a)** TNFα, **b)** IL-1β and **c)** IL-6 in wt mice that received LPS and were either non-treated or treated with rupintrivir. Data are shown as mean ± s.d., with individual data points (n = 5 mice per group). Statistical significance was calculated by means of Welch's two-tailed t-test, *$P < 0.05$, **$P < 0.01$, ***$P < 0.001$, ****$P < 0.0001$, ns, not significant.

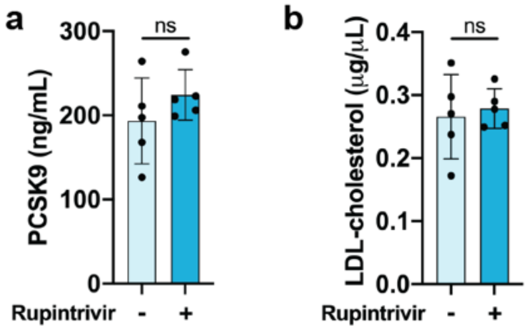

Figure S15: Effect of rupintrivir on PCSK9 and LDL-cholesterol *in vivo*. Blood levels of **a)** PCSK9 and **b)** LDL-cholesterol in WT mice that were either non-treated or treated with rupintrivir. Data are shown as mean ± s.d., with individual data points (n = 5 mice per group). Statistical significance was calculated by means of Welch's two-tailed t-test, *$P < 0.05$, **$P < 0.01$, ***$P < 0.001$, ****$P < 0.0001$, ns, not significant.

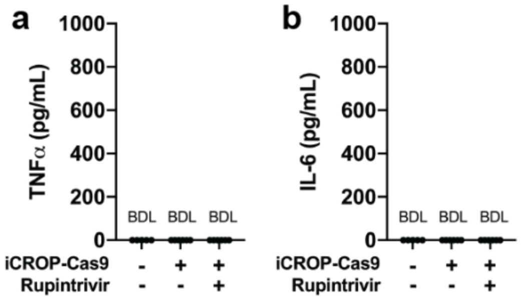

Figure S16: Inflammatory cytokine levels in mice treated with iCROP-Cas9. Blood levels of **a)** TNFα and **b)** IL-6 in mice that were hydrodynamically injected with iCROP-Cas9 and either non-treated or treated with rupintrivir. Data are shown as mean ± s.d., with individual data points (n = 5 mice per group), BDL; below detection level. Statistical significance was calculated by means of Welch's two-tailed t-test, *$P < 0.05$, **$P < 0.01$, ***$P < 0.001$, ****$P < 0.0001$, ns, not significant.

References

1. Haellman, V., Strittmatter, T., Bertschi, A., Stücheli, P. & Fussenegger, M. A versatile plasmid architecture for mammalian synthetic biology (VAMSyB). *Metab. Eng.* **66**, 41–50 (2021).
2. Ausländer, D. *et al.* Programmable full-adder computations in communicating three-dimensional cell cultures. *Nat. Methods* **15**, 57–60 (2018).
3. Ausländer, S., Fuchs, D., Hürlemann, S., Ausländer, D. & Fussenegger, M. Engineering a ribozyme cleavage-induced split fluorescent aptamer complementation assay. *Nucleic Acids Res.* **44**, 94 (2016).
4. Liu, Y. *et al.* Immunomimetic Designer Cells Protect Mice from MRSA Infection. *Cell* **174**, 259-270.e11 (2018).
5. Xie, M. *et al.* β-cell-mimetic designer cells provide closed-loop glycemic control. *Science* **354**, 1296–1301 (2016).
6. Krawczyk, K. *et al.* Electrogenetic cellular insulin release for real-time glycemic control in type 1 diabetic mice. *Science* **368**, 993–1001 (2020).
7. Krawczyk, K., Scheller, L., Kim, H. & Fussenegger, M. Rewiring of endogenous signaling pathways to genomic targets for therapeutic cell reprogramming. *Nat. Commun.* **11**, 1–9 (2020).

8. Sedlmayer, F., Hell, D., Müller, M., Ausländer, D. & Fussenegger, M. Designer cells programming quorum-sensing interference with microbes. *Nat. Commun.* **9**, 1822 (2018).
9. Franko, N., Teixeira, A. P., Xue, S., Charpin-El Hamri, G. & Fussenegger, M. Design of modular autoproteolytic gene switches responsive to anti-coronavirus drug candidates. *Nat. Commun.* **12**, 6786 (2021).
